# Vascular Remodeling of Clinically Used Patches and Decellularized Pericardial Matrices Recellularized with Autologous or Allogeneic Cells in a Porcine Carotid Artery Model

**DOI:** 10.3390/ijms23063310

**Published:** 2022-03-18

**Authors:** Jaroslav Chlupac, Roman Matejka, Miroslav Konarik, Robert Novotny, Zuzana Simunkova, Iveta Mrazova, Ondrej Fabian, Milan Zapletal, Zdenek Pulda, Jan Falk Lipensky, Jana Stepanovska, Karel Hanzalek, Antonin Broz, Tomas Novak, Alena Lodererova, Ludek Voska, Theodor Adla, Jiri Fronek, Miroslav Rozkot, Serhiy Forostyak, Peter Kneppo, Lucie Bacakova, Jan Pirk

**Affiliations:** 1 Transplantation Surgery Department, Institute for Clinical and Experimental Medicine (IKEM), Videnska 1958/9, 14021 Prague, Czech Republic; robert.novotny@ikem.cz (R.N.); jiri.fronek@ikem.cz (J.F.); 2 Department of Biomaterials and Tissue Engineering, Institute of Physiology of the Czech Academy of Sciences, Videnska 1083, 14220 Prague, Czech Republic; antonin.broz@fgu.cas.cz (A.B.); lucie.bacakova@fgu.cas.cz (L.B.); 3 Department of Anatomy, Second Faculty of Medicine, Charles University, V Uvalu 84, 15006 Prague, Czech Republic; tomas.novak@lfmotol.cuni.cz; 4 Department of Biomedical Technology, Faculty of Biomedical Engineering, Czech Technical University in Prague, Nam. Sitna 3105, 27201 Kladno, Czech Republic; roman.matejka@fbmi.cvut.cz (R.M.); jana.stepanovska@fbmi.cvut.cz (J.S.); karel.hanzalek@fbmi.cvut.cz (K.H.); kneppo@fbmi.cvut.cz (P.K.); 5 Cardiovascular Surgery Department, Institute for Clinical and Experimental Medicine (IKEM), Videnska 1958/9, 14021 Prague, Czech Republic; miroslav.konarik@ikem.cz (M.K.); jan.pirk@ikem.cz (J.P.); 6 Institute of Physiology, First Faculty of Medicine, Charles University, Albertov 5, 12800 Prague, Czech Republic; 7 Experimental Medicine Centre, Institute for Clinical and Experimental Medicine (IKEM), Videnska 1958/9, 14021 Prague, Czech Republic; zuzana.simunkova@ikem.cz (Z.S.); iveta.mrazova@ikem.cz (I.M.); 8 Clinical and Transplant Pathology Centre, Institute for Clinical and Experimental Medicine (IKEM), Videnska 1958/9, 14021 Prague, Czech Republic; ondrej.fabian@ikem.cz (O.F.); alena.lodererova@ikem.cz (A.L.); ludek.voska@ikem.cz (L.V.); 9 Radiodiagnostic and Interventional Radiology Department, Institute for Clinical and Experimental Medicine (IKEM), Videnska 1958/9, 14021 Prague, Czech Republic; milan.zapletal@ikem.cz (M.Z.); zdenek.pulda@ikem.cz (Z.P.); theodor.adla@ikem.cz (T.A.); 10 PrimeCell Bioscience, a.s., Dr. Slabihoudka 6232/11, Poruba, 70800 Ostrava, Czech Republic; jan.lipensky@primecell.eu (J.F.L.); serhiy.forostyak@primecell.eu (S.F.); 11 Department of Pig Breeding, Institute of Animal Science, Komenskeho 1239, 51741 Kostelec nad Orlici, Czech Republic; rozkot.miroslav@vuzv.cz

**Keywords:** blood vessel prosthesis, decellularization, cell seeding, tissue engineering, allograft, heterograft, pericardium, adipose tissue-derived stromal cells, Wharton’s jelly mesenchymal stem cells

## Abstract

Background: Cardiovascular surgery is confronted by a lack of suitable materials for patch repair. Acellular animal tissues serve as an abundant source of promising biomaterials. The aim of our study was to explore the bio-integration of decellularized or recellularized pericardial matrices in vivo. Methods: Porcine (allograft) and ovine (heterograft, xenograft) pericardia were decellularized using 1% sodium dodecyl sulfate ((1) Allo-decel and (2) Xeno-decel). We used two cell types for pressure-stimulated recellularization in a bioreactor: autologous adipose tissue-derived stromal cells (ASCs) isolated from subcutaneous fat of pigs ((3) Allo-ASC and (4) Xeno-ASC) and allogeneic Wharton’s jelly mesenchymal stem cells (WJCs) ((5) Allo-WJC and (6) Xeno-WJC). These six experimental patches were implanted in porcine carotid arteries for one month. For comparison, we also implanted six types of control patches, namely, arterial or venous autografts, expanded polytetrafluoroethylene (ePTFE Propaten^®^ Gore^®^), polyethylene terephthalate (PET Vascutek^®^), chemically stabilized bovine pericardium (XenoSure^®^), and detoxified porcine pericardium (BioIntegral^®^ NoReact^®^). The grafts were evaluated through the use of flowmetry, angiography, and histological examination. Results: All grafts were well-integrated and patent with no signs of thrombosis, stenosis, or aneurysm. A histological analysis revealed that the arterial autograft resembled a native artery. All other control and experimental patches developed neo-adventitial inflammation (NAI) and neo-intimal hyperplasia (NIH), and the endothelial lining was present. NAI and NIH were most prominent on XenoSure^®^ and Xeno-decel and least prominent on NoReact^®^. In xenografts, the degree of NIH developed in the following order: Xeno-decel > Xeno-ASC > Xeno-WJC. NAI and patch resorption increased in Allo-ASC and Xeno-ASC and decreased in Allo-WJC and Xeno-WJC. Conclusions: In our setting, pre-implant seeding with ASC or WJC had a modest impact on vascular patch remodeling. However, ASC increased the neo-adventitial inflammatory reaction and patch resorption, suggesting accelerated remodeling. WJC mitigated this response, as well as neo-intimal hyperplasia on xenografts, suggesting immunomodulatory properties.

## 1. Introduction

Patch repair is an essential surgical technique used in cardiac and vascular surgery [1]. In vascular surgery, it comprises patch angioplasty, i.e., patch closure of a longitudinal arteriotomy (with or without endarterectomy for steno-occlusive disease), such as in a carotid, femoral, popliteal, or iliac artery, or in a previous bypass or hemodialysis access graft. Patching may also be used for arteriotomy closure after thrombectomy or embolectomy procedures or during surgery for venous disease. Patches in cardiac surgery are used in the repair of bioprosthetic valve leaflets, intra-cardiac septal defects, aneurysms or ruptures of the heart wall, and reconstructions of the aorta, pulmonary artery, or vena cava [2]. Vessels affected with malignancy may necessitate patch repair within the scope of onco-vascular surgery. When dealing with vascular trauma patients, shorter operative times are required to facilitate resuscitation procedures. Harvesting an autologous vein graft can lead to undesirable prolongation of the surgery. Therefore, an alternative and readily available biological material would be a favorable solution for vascular patch repair in a trauma setting and infected field [3].

The use of patches outside of cardiovascular surgery can be seen in general surgery (abdominal wall and hernia repair), thoracic surgery (suture line reinforcement during lung volume reduction, diaphragm repair), urology, and ophthalmology [1]. 

Selecting a suitable patch material poses a clinical challenge. Current evidence shows roughly equivalent outcomes between autologous, synthetic, and biologic materials. An optimal choice remains unclear, and cardiovascular surgery is in need of novel biomaterials to repair diseased heart valves or blood vessels.

### 1.1. Currently Available Materials for Cardiovascular Repair

Autologous blood vessel replacements generally comprise arterial or venous autografts, i.e., the patient’s own vessels, such as the internal mammary artery, the radial artery, or the great saphenous vein. Autologous vessels represent gold standard materials, boasting the best long-term patency [4]. Their availability, however, is quite limited, and the retrieval of an autograft requires other distant surgical incision(s), usually on the extremities, putting the patient at risk for donor site morbidities such as infection, hematoma, pain, or scarring. Autologous pericardium is utilized in patch repair of intra-cardiac defects in its native or intraoperatively fixed form; however, it may be subject to retraction, thickening, or calcification [5].

Alternatively, synthetic vascular prostheses are widely used. Two primary materials are currently in clinical use: expanded polytetrafluoroethylene (ePTFE, GoreTex^®^, Teflon^®^) and polyester or polyethylene terephthalate (PET, Dacron^®^) [4]. They are readily available and durable. However, they often fail due to diameter and compliance mismatches between the rigid prosthesis and the more elastic native vessel, which may lead to thrombosis on an artificial surface lacking a natural endothelial lining, formation of pseudoaneurysms, and neo-intimal hyperplasia. Vascular prostheses are more prone to infection than biological materials, disqualifying them from use in an infected field [6].

Allograft vessels from cadaveric donors (cold-stored or cryopreserved in a tissue bank) may also be utilized for grafting. Nevertheless, these conduits are not readily available for emergent surgery. Their long-term performance is limited [7], and they burden the recipient with immune suppression therapy, which is required to prevent allograft rejection [8].

Xenograft pericardial tissues are frequently used as biological patches in cardiovascular surgery. The advantages of currently used biological patches include immediate availability, superior biocompatibility, ease of handling, decreased bleeding along the suture line, reduced risk for infection, and the potential for remodeling [1].

**Figure 1:**
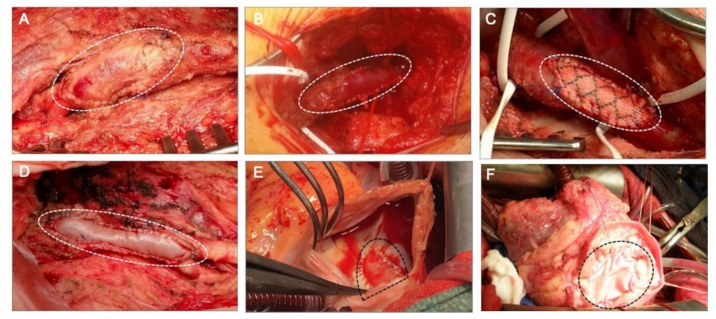
Clinically implanted materials for patch angioplasty. (**A**) Autologous arterial patch used for arteriotomy closure after an endarterectomy of the common and profunda femoral arteries. The patch was created through the harvesting and desobliterating of a chronically occluded superficial femoral artery. (**B**) Autovenous patch created from a saphenous vein graft used for repair of a stenotic distal anastomosis of a femoral–popliteal bypass graft on the popliteal artery. (**C**) Synthetic prosthetic patch made of polyethylene terephthalate (PET) used for patch repair of a stenotic common femoral artery. (**D**) Biologic patch angioplasty of a common femoral artery bifurcation carried out with a xenograft glutaraldehyde-treated bovine pericardium (XenoSure^®^). (**E**) Patch closure of a cardiac interatrial septal defect with an autologous pericardium intraoperatively fixed with formaldehyde. (**F**) Patch closure of a cardiac interventricular septal defect with a xenograft glutaraldehyde-fixed bovine pericardium treated with a proprietary anti-calcification procedure (SJM^®^ Pericardial patch with EnCap^®^ Technology). The patches are marked with dotted lines.

Chemical stabilization of human allografts (homografts) or animal xenografts (heterografts), e.g., cross-linking with glutaraldehyde (GA), as is used in commercially fabricated heart valves [9], vascular grafts [10], and pericardial patches [1], prevents immune responses and degradation. The main advantage of using GA-fixed human allografts is that they are readily available in various sizes; moreover, they do not require immune suppression therapy since the cellular antigens are hidden. Glutaraldehyde treatment, however, elicits altered biomechanics, chronic inflammatory responses, calcifications, and degeneration in the long term due to residual toxicity and cell residues [11]. Several alternative stabilizing procedures have been proposed to minimize the cytotoxicity and calcification of bioprostheses. These would include cross-linking with low concentrations of GA or alternative agents (such as carbodiimide [12] or genipin) or detoxification procedures [13]. Cross-linking, however, is believed to bring about the obstruction of cell–matrix interactions, resulting in tissue encapsulation and the prevention of constructive remodeling [14]. Examples of clinically used materials for patch repair in cardiac and vascular surgery are given in Figure 1. 

Decellularizing native tissue removes the cell antigenicity while preserving the extracellular matrix with the desired microstructure and biomechanical properties. Decellularized non-vascular tissues, such as amniotic membranes, small intestinal submucosa, or pericardial tissue, can also be conditioned for cardiovascular applications [15]. Fixation methods have also been applied to decellularized matrices in order to promote biostability. Examples of these common proprietary procedures include dye-mediated photo-oxidation [16,17,18,19] and glutaraldehyde-based ADAPT^®^ technology [5,20,21,22]. However, residual immunogenicity, incomplete cell removal, and chronic inflammatory responses remain a problem in decellularized tissues [23].

### 1.2. Tissue Engineering Solutions

Tissue-engineered vascular patches may overcome shortcomings seen in currently available materials. They have the ability to grow and regenerate into vascularized tissue. The tissue-engineered acellular matrices of either allograft or xenograft origin have become popular substitutes [24,25]. Two mainstream approaches in tissue engineering involve the implantation of cell-free [26] or cell-seeded constructs [27,28,29,30,31]. Recellularization has been adopted to improve vascular remodeling of decellularized matrices in vivo [32]. The seeded cells attract the ingrowing host cells through a paracrine mechanism, suppress the formation of intimal hyperplasia, and facilitate remodeling of the seeded graft into a neo-vessel [33,34].

Autologous or allogeneic cell types have been used as a cell source in various tissue engineering settings. The utilization of autologous cells, either differentiated (e.g., endothelium from a vein biopsy) or progenitor (e.g., from adipose tissue or bone marrow), burdens the patient with the harvesting procedure, requires a prolonged culture period [35,36], poses a challenge for sterility and storage, and is labor and cost-intensive [15]. Allogeneic immune-privileged cells, such as Wharton’s jelly mesenchymal stem cells (WJCs), could serve as a desirable option [37,38]. They are easily harvested from umbilical cords, pose no ethical issues, are not subject to rejection, and possess excellent proliferation potential, multipotency, and immunosuppressive and immunomodulatory properties [39,40,41]. Moreover, WJCs have both endothelial [42] and smooth muscle cell [43] differentiation potential.

This study aimed to investigate the biological responses and tissue regeneration patterns of clinically used vascular patches (autologous, prosthetic, and bioprosthetic) and experimental allograft and xenograft pericardial patches (decellularized or recellularized individually with two cell types) from the perspective of potential application in cardiovascular tissue engineering and surgery. The patches were implanted to repair a defect in porcine carotid arteries and left in place for one month. We made a concerted effort to cover and compare a wide range of materials and, therefore, implanted six clinically used vascular patches as control grafts. These comprised the following: two types of autologous patches, namely arterial autografts and venous autografts; and four types of commercially available materials, which comprised two types of prosthetic patches, namely ePTFE (Propaten^®^ Gore^®^) and PET (Vascutek^®^), and two types of bioprosthetic patches, namely bovine pericardium chemically stabilized with glutaraldehyde (XenoSure^®^) and detoxified porcine pericardium (BioIntegral^®^ NoReact^®^). We further implanted six types of experimental pericardial patches. These comprised decellularized porcine (allograft) and ovine (xenograft) patches (Allo-decel and Xeno-decel, respectively), recellularized allograft and xenograft patches with autologous adipose tissue-derived stromal cells (Allo-ASC and Xeno-ASC, respectively), and recellularized allograft and xenograft patches with WJCs (Allo-WJC and Xeno-WJC, respectively). A list of the control and experimental patch implants is presented in Table 1. 

## 2. Results

### 2.1. Experimental Pericardial Pacthes

#### 2.1.1. Decellularization

**Figure 2:**
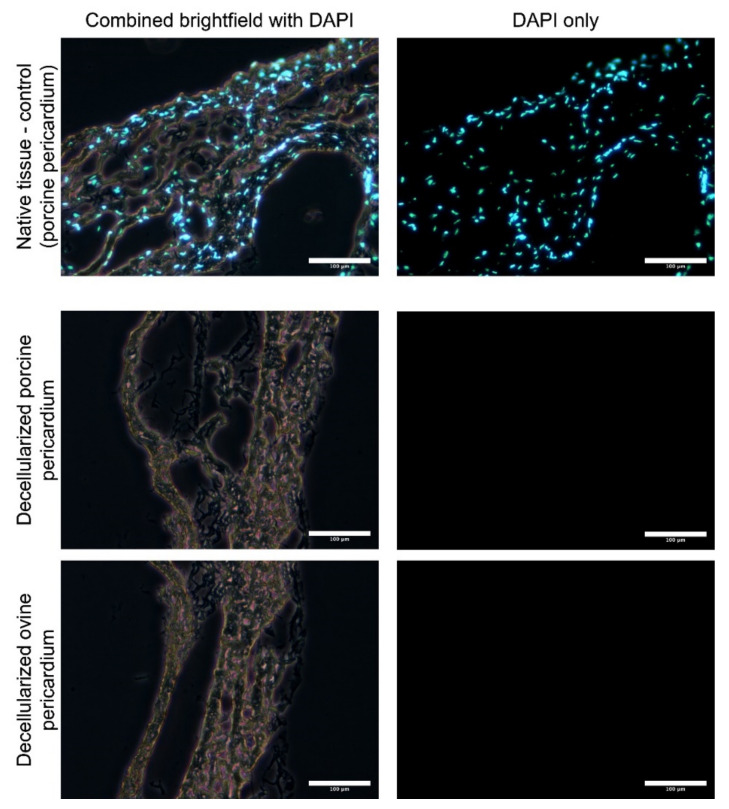
Thin cryosections (7 μM) of native and decellularized porcine and ovine pericardia stained with DAPI. Cell nuclei are visible in native tissue; however, neither nuclei nor fragments were observed in the decellularized pericardia.

Decellularized porcine allograft and ovine xenograft pericardia were prepared as substrates for further recellularization with porcine autologous ASCs or allogenous WJCs. After decellularization in an automated system and sterilization in an ethanol solution, the tissues were analyzed for the remaining cells. Thin cryosections (7 μm) were made and samples were stained with DAPI. As illustrated in Figure 2, there are no visible cell nuclei or fragments of cell nuclei in decellularized tissues.

#### 2.1.2. Recellularization

**Figure 3:**
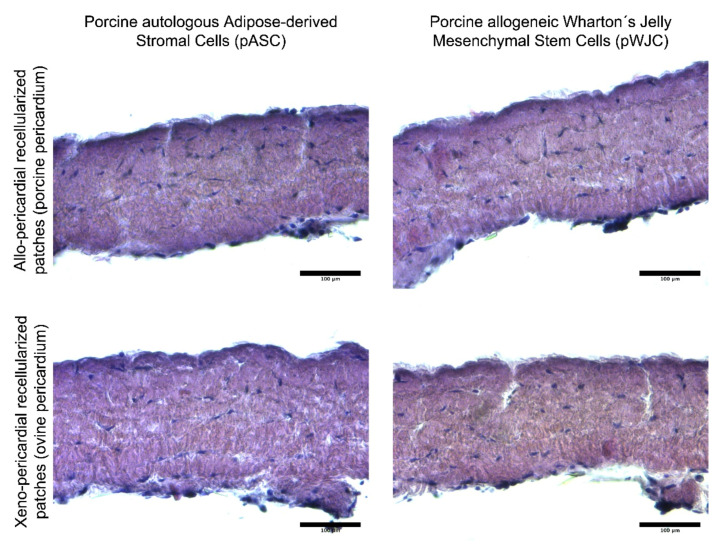
Histological evaluation (paraffin-embedded sections stained with hematoxylin and eosin) of bioreactor-processed implantable patches based on decellularized porcine and ovine pericardia. Newly seeded cells penetrated the entire thickness of decellularized tissue after being cultivated in dynamic conditions for 5 days.

Recellularized pericardial patches were histologically analyzed after cultivation in a bioreactor (prior to implantation). As illustrated in Figure 3, both ASCs and WJCs show signs of ingrowth into the decellularized porcine and ovine pericardia. After 5 days of dynamic culture, the entire thickness of decellularized tissue is recellularized with new cells.

### 2.2. Surgical Experiment In Vivo

#### 2.2.1. Implantation

The mean surgery time was 126 ± 20 min, while the mean carotid artery clamping time was 22 ± 5 min. There was very little difference between the clamping time of the control patches (22 ± 6 min) and that of the pericardial patches (23 ± 3 min, *p* = 0.444, nonsignificant (n.s.)), which indicates efficient surgical handling of the experimental pericardia.

#### 2.2.2. Flowmetry

In the control patches, the mean blood flow in the native artery prior to implantation was 242 ± 92 mL/min at a mean arterial pressure (MAP) of 73 ± 8 mmHg. The flow after implantation markedly dropped to 144 ± 109 mL/min (*p* = 0.056, n.s.) at an MAP of 68 ± 8 mmHg. This drop was brought on by spasms of the muscular elastic porcine carotid arteries. The mean flow at explantation was 189 ± 129 mL/min (*p* = 0.420 vs. post-implantation, n.s.) at an MAP of 79 ± 12 mmHg.

**Figure 4:**
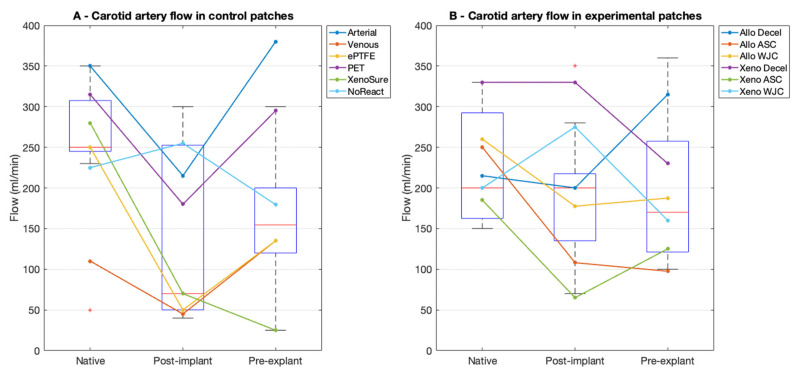
Flowmetry results in porcine carotid arteries. (**A**) We implanted six types of control vascular patches and (**B**) six types of experimental pericardial porcine allograft and ovine xenograft patches in a decellularized or recellularized form. Red lines represent median values, blue boxes 1st and 3rd quartiles, whiskers the minimum and maximum, and the red circles outliers.

In the experimental patches, the mean flow in the native artery prior to implantation was 232 ± 112 mL/min at an MAP of 63 ± 10 mmHg. The flow after implantation also dropped markedly, albeit not significantly, to 180 ± 99 mL/min (*p* = 0.265) at an MAP of 67 ± 10 mmHg. The mean flow at explantation was 182 ± 101 mL/min (*p* = 0.968 vs. post-implantation, n.s.) at an MAP of 86 ± 15 mmHg. The flowmetry is presented in Figure 4. 

#### 2.2.3. Angiography

**Figure 5:**
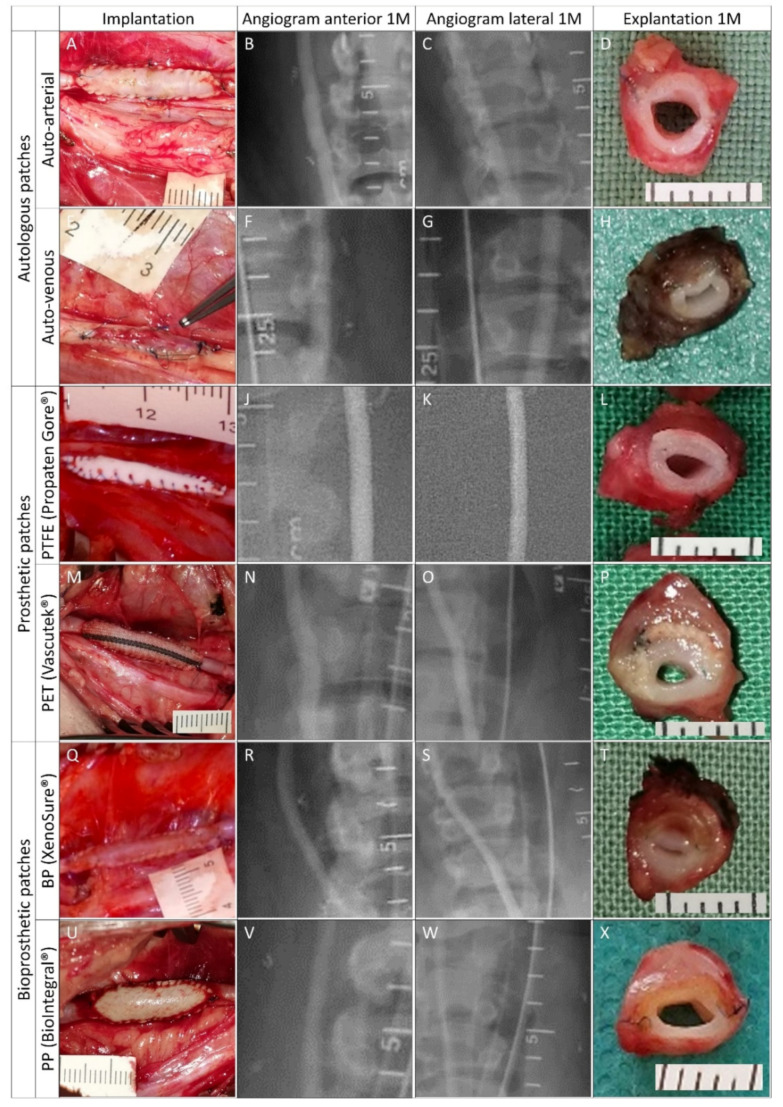
Clinically used vascular patches in porcine carotid arteries: implantation, pre-explant angiography, and explantation. We implanted two types of autologous patches: (**A**–**D**) arterial autograft and (**E**–**H**) venous autograft; two types of prosthetic patches: (**I**–**L**) expanded polytetrafluoroethylene (ePTFE, Propaten^®^ Gore^®^) and (**M**–**P**) polyethylene terephthalate (PET, Vascutek^®^); and two types of bioprosthetic patches: (**Q**–**T**) bovine pericardium chemically stabilized with glutaraldehyde (BP, XenoSure^®^) and (**U**–**X**) detoxified porcine pericardium (PP, BioIntegral^®^ NoReact^®^). Macroscopic views of the patches after declamping and hemostasis during implantations are shown in the left-hand column. Selective carotid angiograms performed from groin access at 1 month (1 M) post-implantation are shown in the middle two columns (anterior–posterior and lateral projections). The boundaries of the implanted patch are marked with metal clips. Gross appearances of cross-sectioned explants at 1 M are presented in the right-hand column. Implanted patches comprise the upper half of the vessel. Since the angiograms turned out normal (R and S), the apparent lumen loss in T was probably due to spasms during explantation.

**Figure 6:**
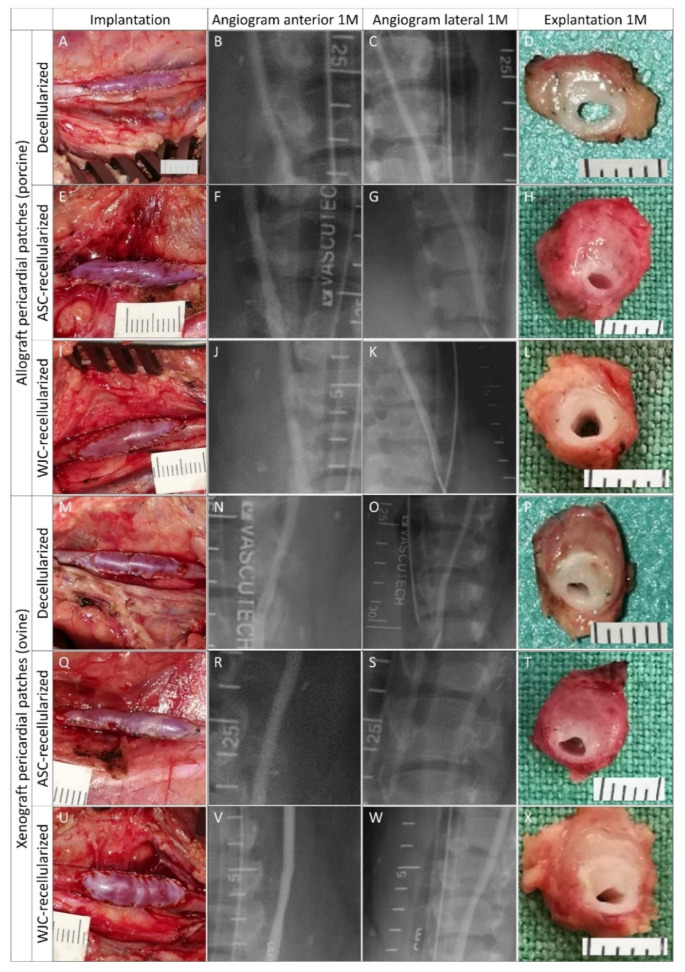
Experimental pericardial vascular patches in porcine carotid arteries: implantation, pre-explant angiography, and explantation. We implanted decellularized porcine allograft and ovine xenograft patches ((**A**–**D**) and (**M**–**P**), respectively), allograft and xenograft patches recellularized with autologous adipose tissue-derived stromal cells (ASCs, (**E**–**H**) and (**Q**–**T**), respectively), and allograft and xenograft patches recellularized with allogeneic Wharton’s jelly mesenchymal stem cells (WJCs; (**I**–**L**) and (**U**–**X**), respectively). Macroscopic views of the patches after declamping and hemostasis during implantation are shown in the left-hand column. Selective carotid angiograms performed from groin access at 1 month (1 M) post-implantation are shown in the middle two columns (anterior–posterior and lateral projection). The boundaries of the implanted patch are marked with metal clips. Gross appearances of cross-sectioned explants at 1 M are presented in the right-hand column. Implanted patches comprise the upper half of the vessel.

Pre-explant selective angiography of all carotid arteries in anteroposterior and lateral projections revealed favorable clinical outcomes at 1 month post-implantation. There were no signs of stenosis or aneurysm formation (Figure 5 and Figure 6). Regarding patency or luminal area assessment, we consider angiography the most powerful method. Unlike flowmetry, angiography was not adversely affected by vasospasms due to surgical dissection, and unlike histology, it was not affected by potential shrinkage due to tissue processing. 

#### 2.2.4. Explantation

Gross examination of all of the explanted specimens showed good integration and no thrombosis. Graft infection did not occur. Angiograms, intraoperative and macroscopic views of control explants, and experimental patches are shown in Figure 5 and Figure 6. Since the angiograms were normal (Figure 5, R and S), the apparent loss of lumen in the XenoSure^®^ explant (Figure 5T) was most likely due to spasms during explantation.

### 2.3. Microscopical Examination

#### 2.3.1. Histology and Immunohistochemistry

- Control Patches

**Figure 7:**
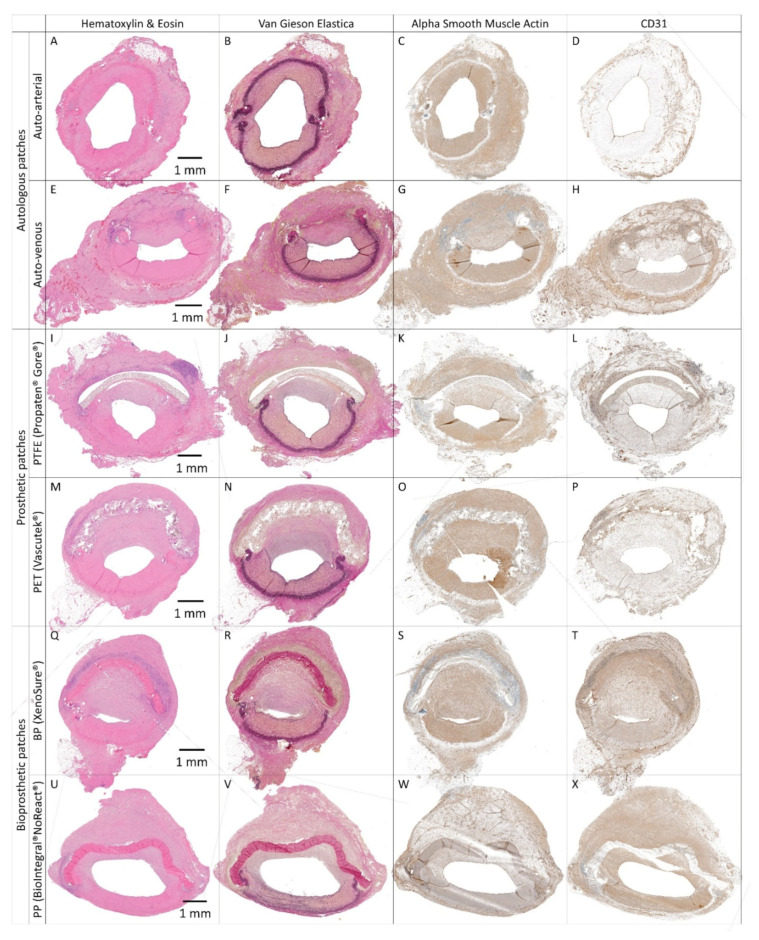
Histology and immunohistochemistry of control vascular patches in porcine carotid arteries 1 month post-implantation. We implanted two types of autologous patches: (**A**–**D**) arterial autografts and (**E**–**H**) venous autografts; two kinds of prosthetic patches: (**I**–**L**) expanded polytetrafluoroethylene (ePTFE, Propaten^®^ Gore^®^) and (**M**–**P**) polyethylene terephthalate (PET, Vascutek^®^); and two types of bioprosthetic patches: (**Q**–**T**) bovine pericardium (BP) chemically stabilized with glutaraldehyde (XenoSure^®^) and (**U**–**X**) detoxified porcine pericardium (PP, BioIntegral^®^ NoReact^®^). Representative cross-sections of the midgraft regions are shown. Implanted patches comprise the upper half of the vessel, magnification 20×. The apparent lumen loss in XenoSure^®^ (**Q**–**T**) was probably due to spasms during explantation.

**Figure 8:**
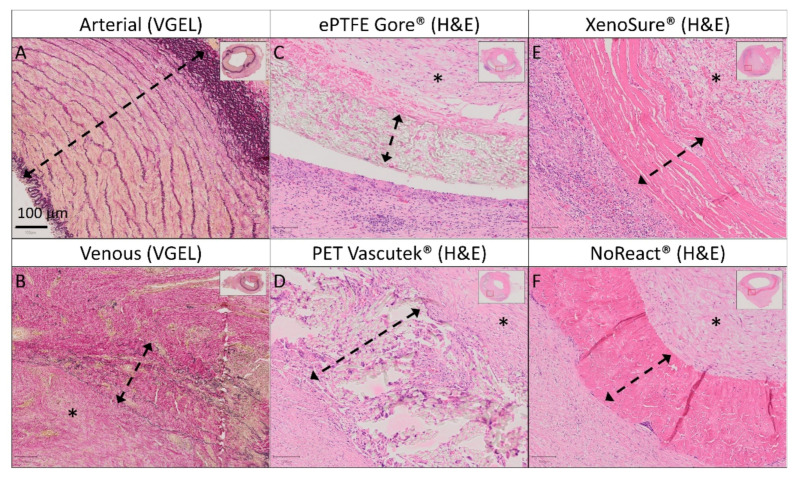
Detailed histology of control vascular patches in porcine carotid arteries 1 month post-implantation. We implanted two types of autologous patches: (**A**) arterial autografts and (**B**) venous autografts; two kinds of prosthetic patches: (**C**) expanded polytetrafluoroethylene (ePTFE, Propaten^®^ Gore^®^) and (**D**) polyethylene terephthalate (PET, Vascutek^®^); and two types of bioprosthetic patches: (**E**) bovine pericardium (BP) chemically stabilized with glutaraldehyde (XenoSure^®^) and (**F**) detoxified porcine pericardium (PP, BioIntegral^®^ NoReact^®^). Representative cross-sections of the midgraft regions are shown. We performed staining with hematoxylin and eosin (H&E) and Van Gieson Elastica. A double arrow denotes the implanted patch material, and an asterisk denotes the luminal side of the patch and neo-intimal tissue (magnification 20×).

Representative histological and immunohistochemical examinations of midgraft cross-sections of the control patches are presented in Figure 7 and Figure 8. Full size images are given in Supplementary Figures S1–S6. All patches were well integrated. The arterial autograft (Figure 7A–D and Figure 8A) resembled a native artery, had a well-preserved layered structure and no signs of neo-intimal hyperplasia (NIH), and may serve as a positive control. All other control implants induced the formation of NIH on the luminal surface of the patch. The NIH was composed of a proliferation of fibroblasts and myofibroblasts. The luminal endothelial lining was present in all patches. In some samples, the lining was discontinuous and most likely partially stripped during tissue processing.

Venous autografts (Figure 7E–H and Figure 8B) induced perigraft fibrotization, hyalinization, a giant cell reaction, and mild chronic lymphoplasmacytic inflammatory cellularization. 

The ePTFE Gore^®^ (Figure 7I–L and Figure 8C) and PET Vascutek^®^ (Figure 7M–P and Figure 8D) prosthetic patches similarly produced diffuse neo-adventitial fibrosis, a giant cell reaction, and a chronic lymphoplasmacytic inflammatory reaction. Slight separation of the ePTFE patch from surrounding tissues occurred artificially.

XenoSure^®^ patches (Figure 7Q–T and Figure 8E) provoked a giant cell reaction, focal calcifications, and a chronic mixed inflammatory reaction, including numerous neutrophils. The neo-adventitia was diffusely fibrotic. In addition to substantial NIH in the patch lumen, diffuse mild intimal fibrosis was also present in the opposite lumen of the native vessel. The apparent lumen loss was probably due to spasms during explantation. 

NoReact^®^ patches (Figure 7U–X and 8F) also elicited a giant cell reaction and diffuse neo-adventitial fibrosis but evoked only minimal focal chronic inflammation. Luminal NIH was mild, and light intimal fibrous thickening also appeared in the opposite native vessel.

- Experimental pericardial patches

**Figure 9:**
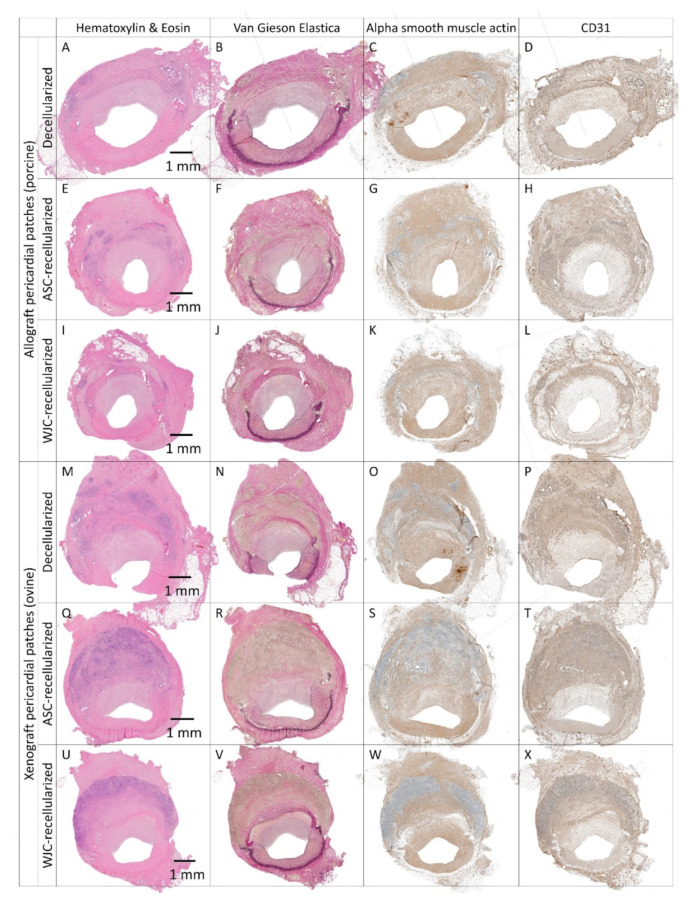
Histology and immunohistochemistry of experimental pericardial vascular patches in porcine carotid arteries 1 month post-implantation. We implanted decellularized (**A**–**D**) porcine allograft and (**M**–**P**) ovine xenograft patches, (**E**–**H**) allograft and (**Q**–**T**) xenograft patches recellularized with autologous adipose tissue-derived stromal cells (ASCs), and (**I**–**L**) allograft and (**U**–**X**) xenograft patches recellularized with allogeneic Wharton’s jelly mesenchymal stem cells (WJCs). Representative cross-sections of the midgraft regions are shown. Implanted patches comprise the upper half of the vessel, magnification 20×.

**Figure 10:**
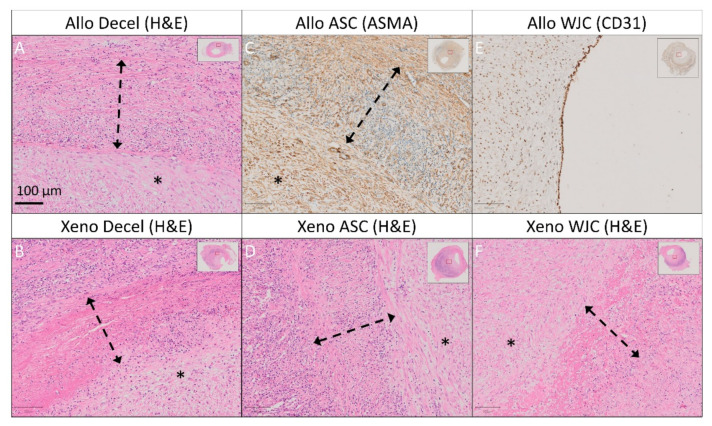
Detailed histology and immunohistochemistry of experimental pericardial vascular patches in porcine carotid arteries 1 month post-implantation. We implanted decellularized (**A**) porcine allograft and (**B**) ovine xenograft patches, (**C**) allograft and (**D**) xenograft patches recellularized with autologous adipose tissue-derived stromal cells (ASCs), and (**E**) allograft and (**F**) xenograft patches recellularized with allogeneic Wharton’s jelly mesenchymal stem cells (WJCs). Representative cross-sections of the midgraft regions are shown. We performed staining with hematoxylin and eosin (H&E) and immunohistochemistry of alpha-smooth muscle actin (ASMA) and endothelial CD31. A double arrow denotes the implanted patch material, and an asterisk denotes the luminal side of the patch and neo-intimal tissue (magnification 20×).

Representative histological and immunohistochemical examinations of midgraft cross-sections of the experimental pericardial patches are presented in Figure 9 and Figure 10. Full size images are given in Supplementary Figures S7–S12. All patches were well integrated. As was the case with most of the control patches, all experimental implants induced the formation of NIH on the luminal surface of the patch. The NIH was composed of a proliferation of fibroblasts and myofibroblasts. The luminal endothelial lining was present in all patches. In some samples, the lining was discontinuous and most likely partially stripped during tissue processing. 

Allo-decel (Figure 9A–D and Figure 10A) induced a giant cell reaction, chronic lymphoplasmacytic inflammatory cellularization, neo-adventitial fibrosis, minor calcifications, and light intimal fibrosis in the opposite native vessel. The patch material was moderately resorbed. 

Allo-ASC (Figure 9E–H and Figure 10C) also produced a giant cell reaction, chronic lymphoplasmacytic inflammatory cellularization (intensive), neo-adventitial fibrosis, minor calcifications, and light intimal fibrosis of the opposite native vessel. The patch material was markedly resorbed.

Allo-WJC (Figure 9I–L and Figure 10E) similarly provoked a giant cell reaction, chronic lymphoplasmacytic cellularization (less intensive), neo-adventitial fibrosis, minor calcifications, and light intimal fibrosis of the opposite native vessel. The patch material was mildly resorbed.

Xeno-decel (Figure 9M–P and Figure 10B) induced a giant cell reaction, chronic lymphoplasmacytic inflammatory cellularization (intensive with the formation of lymphatic aggregates), neo-adventitial fibrosis, minor calcifications, and light intimal fibrosis in the opposite native vessel. The patch material was moderately resorbed.

Xeno-ASC (Figure 9Q–T and Figure 10D) produced a giant cell reaction, chronic lymphoplasmacytic inflammatory cellularization (highly intensive with the formation of germinal centers), neo-adventitial fibrosis, minor calcifications, and very light intimal fibrosis in the opposite native vessel. The patch material was markedly resorbed. 

Xeno-WJC (Figure 9U–X and Figure 10F) provoked a giant cell reaction, chronic lymphoplasmacytic inflammatory cellularization (intensive with the formation of lymphatic aggregates), neo-adventitial fibrosis, minor calcifications, and light intimal fibrosis in the opposite native vessel. The patch material was mildly resorbed.

#### 2.3.2. Neo-Intimal Hyperplasia Thickness

**Figure 11:**
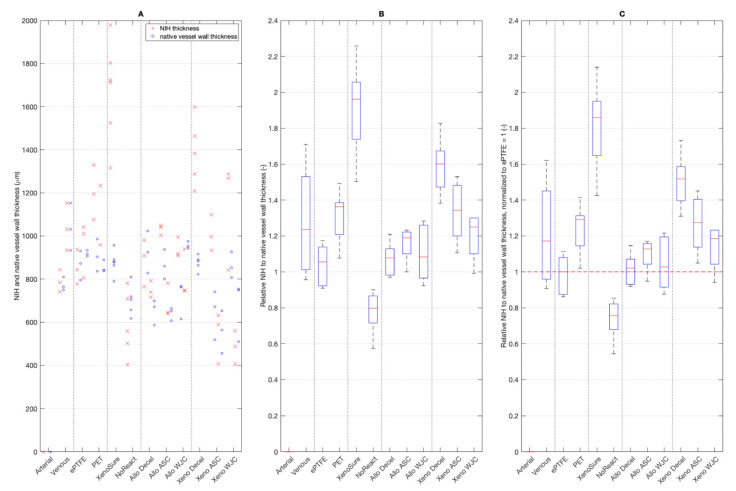
(**A**) Thickness of neo-intimal hyperplasia (NIH) on the control and experimental patches. (**B**) Fold thickness of NIH related to the thickness of the native vessel wall opposite the patch. (**C**) Fold thickness of NIH normalized to that of ePTFE, which was set at 1. Red lines in charts represent median values, blue boxes 1st and 3rd quartiles, and whiskers the minimum and maximum. Note: there was no NIH formation on the arterial autograft. We implanted six types of clinically used vascular patches as control grafts, namely two types of autologous patches: arterial autograft (Arterial) and venous autograft (Venous), two kinds of prosthetic patches: expanded polytetrafluoroethylene (ePTFE, Propaten^®^ Gore^®^) and polyethylene terephthalate (PET, Vascutek^®^), and two types of bioprosthetic patches: bovine pericardium chemically stabilized with glutaraldehyde (XenoSure^®^) and detoxified porcine pericardium (NoReact^®^ BioIntegral^®^). We further implanted six types of experimental pericardial patches, namely decellularized porcine allograft and ovine xenograft patches (Allo-decel and Xeno-decel, respectively), allograft and xenograft patches recellularized with autologous adipose tissue-derived stromal cells (Allo-ASC and Xeno-ASC, respectively), and allograft and xenograft patches recellularized with Wharton’s jelly mesenchymal stem cells (Allo-WJC and Xeno-WJC, respectively).

NIH formed on the luminal surface of all patches with the exception of the arterial autograft. Absolute values of NIH thickness are given in Figure 11A. Fold ratios of NIH thickness related to the thickness of the native vessel wall opposite the patch are shown in Figure 11B. The NIH fold ratios that were normalized to those on ePTFE are presented in Figure 11C. 

Because of the limited number of animals, the results have a large scatter. Thus, the only statistically significant (*p* = 0.05) differences were as follows. Firstly, the NoReact^®^ surface had the lowest NIH thickness compared with any other patch. Secondly, the XenoSure^®^ had the highest NIH value compared with all other patches except for Venous and Xeno-Decel patches. 

On the other hand, the results show trend values. NIH was more pronounced on PET than it was on ePTFE. NIH was the most and least visible on the XenoSure^®^ and NoReact^®^ surfaces, respectively. NIH on all of the allo-porcine surfaces was comparable to that of the ePTFE. In contrast, substantial NIH developed on the Xeno-decel patches. The seeding of ASCs and especially WJCs mitigated the NIH response.

#### 2.3.3. Grading of Neo-Adventitial Inflammation, Patch Resorption, Calcifications, and Neo-Intimal Hyperplasia

**Figure 12:**
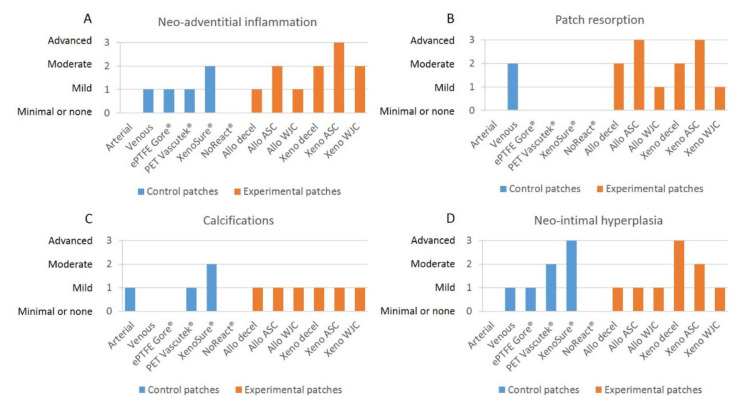
Grades of (**A**) neo-adventitial inflammation, (**B**) patch resorption, (**C**) calcifications, and (**D**) neo-intimal hyperplasia in control and experimental patches. We implanted six types of clinically used vascular patches as control grafts, namely two types of autologous patches: arterial autograft (Arterial) and venous autograft (Venous), two kinds of prosthetic patches: expanded polytetrafluoroethylene (ePTFE, Propaten^®^ Gore^®^) and polyethylene terephthalate (PET, Vascutek^®^), and two types of bioprosthetic patches: bovine pericardium chemically stabilized with glutaraldehyde (XenoSure^®^) and detoxified porcine pericardium (NoReact^®^ BioIntegral^®^). We further implanted six types of experimental pericardial patches, namely decellularized porcine allograft and ovine xenograft patches (Allo-decel and Xeno-decel, respectively), allograft and xenograft patches recellularized with autologous adipose tissue-derived stromal cells (Allo-ASC and Xeno-ASC, respectively), and allograft and xenograft patches recellularized with Wharton’s jelly mesenchymal stem cells (Allo-WJC and Xeno-WJC, respectively).

Grades of neo-adventitial inflammation, patch resorption, calcifications, and neo-intimal hyperplasia in control and experimental patches are presented in Figure 12.

- Neo-adventitial inflammation

There was no inflammatory response to the arterial autograft. The venous autografts, the ePTFE Gore^®^, and the PET Vascutec^®^ induced mild inflammatory infiltration. XenoSure^®^ provoked inflammatory cellularization to a moderate extent. NoReact^®^, on the other hand, produced minimal inflammation (Figure 7).

Allo-decel induced a common mild inflammatory response. Allo-ASC produced moderate inflammation. Allo-WJC brought about a mild-grade inflammatory reaction. Xeno-decel provoked moderate inflammation, xeno-ASC substantial inflammation, and Xeno-WJC a moderate response (Figure 9).

- Patch resorption

The arterial autograft was identical in appearance to a native artery (no resorption). In contrast, the venous autograft showed a moderate degree of resorption. Remnants of elastic fibers were still visible. The materials used in ePTFE Gore^®^, PET Vascutek^®^, XenoSure^®^, and NoReact^®^ patches are non-resorbable. We observed no cell ingrowth into the amorphous ePTFE Gore^®^ patch. Conversely, the PET Vascutek^®^ knitted material permitted cell invasion, which comprised primarily foreign-body giant cells and fibroblasts. Residues of original cell components were noticeable in the biological XenoSure^®^ and NoReact^®^ patches. Furthermore, these two patches became slightly loosened on the periphery and were invaded with newly ingrowing fibrous tissue (Figure 8).

Allo-decel and Xeno-decel were moderately resorbed. Allo-ASC and Xeno-ASC were resorbed to an advanced grade; the Xeno-ASC patch was, in particular, barely visible upon resorption. There was only mild resorption in Allo-WJC and Xeno-WJC (Figure 10).

- Calcifications

In general, very few calcifications occurred. They were slightly apparent in the arterial autograft, the PET Vascutek^®^, and in all experimental pericardial patches. They were moderately noticeable in XenoSure^®^.

- Neo-intimal hyperplasia

Qualitative grading of NIH reflects the above-mentioned quantitative measurements of NIH thickness. 

### 2.4. Hematology

**Figure 13:**
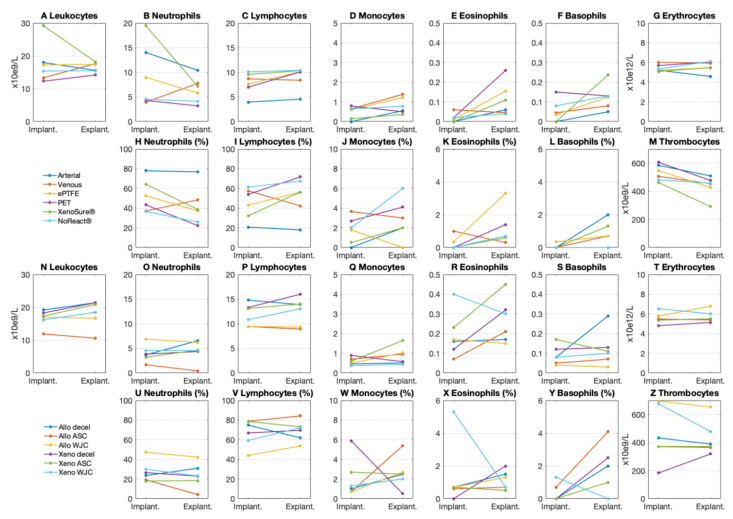
Blood counts in laboratory pigs implanted with (**A**–**M**) control and (**N**–**Z**) experimental patches. We implanted six types of clinically used vascular patches as control grafts, namely two types of autologous patches: arterial autograft (Arterial) and venous autograft (Venous), two kinds of prosthetic patches: expanded polytetrafluoroethylene (ePTFE, Propaten^®^ Gore^®^) and polyethylene terephthalate (PET, Vascutek^®^), and two types of bioprosthetic patches: bovine pericardium chemically stabilized with glutaraldehyde (XenoSure^®^) and detoxified porcine pericardium (NoReact^®^ BioIntegral^®^). We further implanted six types of experimental pericardial patches, namely decellularized porcine allograft and ovine xenograft patches (Allo-decel and Xeno-decel, respectively), allograft and xenograft patches recellularized with autologous adipose tissue-derived stromal cells (Allo-ASC and Xeno-ASC, respectively), and allograft and xenograft patches recellularized with Wharton’s jelly mesenchymal stem cells (Allo-WJC and Xeno-WJC, respectively).

Differential blood counts in laboratory pigs at the time of implantation and explantation are presented in Figure 13. There were no signs of an immune response in the peripheral blood of pigs implanted with control or experimental patches.

## 3. Discussion

This study, involving porcine carotid artery models, confirmed the suitability of pericardial tissues for vascular repair as the patches remained patent one month after surgery. Patency was also observed in the control patches, namely the autologous and commercially available prosthetic and biological patches. The clinical outcome was favorable for all samples. We observed differences in tissue remodeling only through microscopic examinations. It may be assumed that decellularized pericardia repopulated with WJCs provided a few more benefits than did blank tissues or ASC-seeded matrices. Such advantages include the following: no requirement to harvest autologous cells, slight mitigation of neo-adventitial inflammation, and mild suppression of neo-intimal hyperplasia.

### 3.1. Limitations

There are limitations to our study. Firstly, the 1-month implant period may have been too short to evaluate all possible tissue restoration events. We currently have no knowledge of the potential progress or regress of certain conditions, such as NAI, NIH, or clinical patency, beyond one month. 

Secondly, results from a short study in a healthy juvenile laboratory animal can hardly reflect realistic issues seen in a diseased human patient [44]. Therefore, any extrapolation must be made with caution.

**Figure 14:**
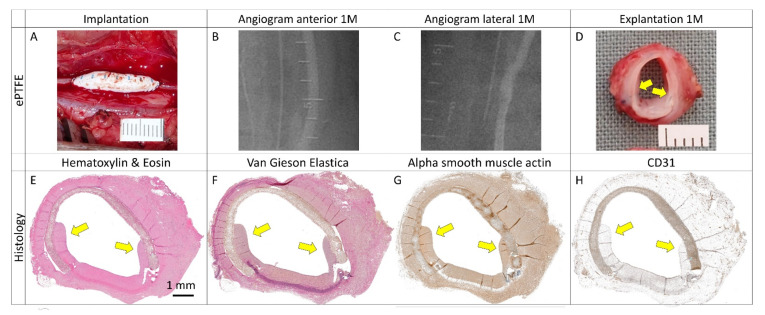
(**A**) Implantation and explantation (**B**–**D**) of an oversized vascular patch in a porcine carotid artery for 1 month (1 M). (**E**–**G**) Trans-anastomotic pannus overgrowth and (**H**) endothelial lining were observed from the native artery to the expanded polytetrafluoroethylene (ePTFE) patch (yellow arrows).

Thirdly, the vascular patch we implanted was relatively small compared with the larger patches or long bypass grafts used in clinical scenarios. Cardiovascular implants repopulate in vivo through three independent or concurrent modalities: trans-anastomotic (TA) outgrowth, trans-mural (TM) ingrowth, or the fallout (FO) of progenitor cells from the blood. Unlike in that of animals, TA and FO modes of graft healing in humans have their limitations [45]. Our supplementary experiment involved implanting an oversized ePTFE Gore^®^ patch that formed a bulge. Examination of retrieved samples showed pannus tissue overgrowing onto the patch lumen from the adjacent vessel. The pannus contained surface endothelium. These results indicate the neo-intimal TA healing typically seen in animal studies (Figure 14 and Supplementary Figure S13). That is why differences between bare, ASC, and WJC-seeded patches might have been blurred due to one predominant TA mode of tissue regeneration. Differentiating TA, TM, and FO modes of healing for our samples would necessitate a more sophisticated animal model of indirect or isolation loop implantation. This model would enable us to separate the implant from adjacent arterial tissue and thus from TA overgrowth [12,46,47].

Finally, we evaluated the inflammatory infiltrates in the perigraft tissue only semi-quantitatively. NAI per se should not be regarded as an adverse event. On the contrary, tissue-engineered grafts commonly transform into a neo-vessel through an inflammation-mediated process. The seeded cells secrete soluble factors that attract cell ingrowth from neighboring tissue and promote homing of circulating monocytes that produce angiogenic factors and further stimulate recruitment of endothelial and smooth muscle cells and regeneration into a blood vessel [33]. We were unable to identify precisely the cell types in the NAI infiltrates, such as pro-inflammatory M1 or anti-inflammatory M2 macrophages [48] or lymphocyte subtypes [49,50,51].

### 3.2. Existing Results

Stöwe et al. [52] noted more favorable host tissue integration, a less pro-inflammatory reaction, and a more improved vascularization pattern of a novel acellular collagen material derived from a porcine aorta compared with XenoSure^®^ in a rat subcutaneous implantation model. Notably, they also observed cell debris in microscopic closeups of XenoSure^®^. Cellular remnants have been linked to adverse tissue reactions [11]. This is roughly in line with our findings, which indicate that this material induced pronounced NAI and NIH after implantation into a vessel; however, the clinical outcome was acceptable. Modern manufacturing processes eliminate low levels of residual glutaraldehyde and any residual cellularity [53]. In our experiment, we chose to implant a more advanced material (NoReact^®^) that was also GA-fixed but treated with a proprietary detoxification process [54]. This material did indeed provoke less prominent NAI and NIH, which was indicated on histological slides. Patch remodeling, however, did not occur since the patch is non-resorbable.

Chang et al. [55] compared acellular bovine pericardium fixed with glutaraldehyde to that fixed with a natural agent (genipin) as pulmonary artery patches in the canine pulmonary trunk. Similarly to our study, they found more pronounced adverse events, such as thicker neo-intima and less endothelial coverage on GA-fixed tissue at 1 month post-implantation. In contrast, our decellularized pericardia were not fixed, and, therefore, they quickly became invaded by host cells, resorbed, and replaced with fibrous tissue. Moreover, resorption was accelerated in ASC-seeded patches.

Cell-seeded BP patches have been previously investigated in animal models. Chang et al. [56] further studied the use of a blank porous genipin-fixed acellular BP, a BP loaded with basic fibroblast growth factor (b-FGF), and a BP populated with b-FGF and mesenchymal stem cells (MSCs) as a patch for repair of a right-ventricular myocardial defect in rats for 1 or 3 months. The extent of tissue regeneration in the b-FGF and b-FGF/MSC patches was more pronounced than that in the control patch. Moreover, labeled cardiomyocytes, smooth muscle cells, and endothelial cells were identified in the b-FGF/MSC patch, while no cardiomyocytes were observed in the control and b-FGF patches. These results showed tissue regeneration within a BP through cell recruitment and tissue-specific differentiation. Our study also observed tissue regeneration in blank decellularized pericardia as well as in ASC or WJC-recellularized pericardia. Although the differences were modest, patch remodeling was slightly accelerated in ASC and slightly modulated in WJC-seeded patches, remotely resembling tissue-specific remodeling.

We et al. [57] implanted an acellular BP with an increased pore size and a BP with labeled MSCs to repair a surgically created myocardial defect in the right ventricle in a syngeneic rat model. Intimal thickening was observed for both studied groups. Endothelial and mesothelial cells were identified on the inner and outer (epicardial) surfaces of the MSC patch. Smooth muscle cells and neo-capillaries were observed within the pores of the MSC-BP. A number of labeled cardiomyocytes were observed in the MSC patch, indicating that the implanted MSCs can engraft and differentiate into cardiomyocytes. Tissue regeneration was not observed for the control patch. The authors concluded that the MSC patch preserved the ventricular structure and provided myocardial tissue regeneration. NIH also developed in all of our studied groups, and the level of NIH was similar in Allo-decell, Allo-ASC, and Allo-WJC. However, seeded cells in xenograft pericardia mitigated the NIH response in the following order: Xeno-decel > Xeno-ASC > Xeno-WJC. This may imply improved vascular remodeling in cell-seeded patches. We did not label the seeded cells, nor did we study cell differentiation post-implantation. The newly formed tissue underneath our patches was composed of fibroblasts and myofibroblasts that were positively stained for alpha-smooth muscle actin.

The benefits of ASC-seeded decellularized scaffolds have previously been demonstrated. Lin et al. [58] compared a bare decellularized porcine coronary artery matrix with that seeded with ASCs in a rat model of aortic patch repair. One month after implantation, the ASC-seeded scaffolds showed better endothelialization and maintained tissue integrity. Similarly to our study, both materials were infiltrated with inflammatory cells and provoked NIH. However, we observed similar endothelialization in all samples of our setting. Additionally, we noted a tendency towards accelerated resorption of the ASC-seeded BP patch and a reduction in NIH thickness in Xeno-ASC. 

Cho et al. [59] fabricated tissue-engineered patches by seeding autologous bone marrow MSCs onto decellularized canine inferior vena cava matrices and implanted them as patches into thoracic inferior vena cava in dogs. A histological analysis revealed regeneration of the endothelium and smooth muscle as well as the presence of collagen and elastin 3 weeks post-implantation. Regrettably, they did not carry out control implantation of the unseeded matrix.

Previous studies mostly applied static methods of cell seeding [60], which provide only surface cell coverage of the bioengineered patches [56,57,58,59]. Significant cell washout may occur after implantation in vivo [34]. Moreover, tissue grafts with a cell population within the entire wall provide better support for remodeling. Chen et al. [61] provided a spatially uniform distribution of adhered cells within a scaffold. They sandwiched a porous acellular BP with multilayered sheets of MSCs. These patches were used to replace infarcted myocardial walls in rats. Sandwich-patch-treated hearts demonstrated enhanced structural support and better heart function than did empty-patch-treated hearts. In our study, we ensured a uniform cellular distribution within the acellular BP scaffold through dynamic seeding; hydrostatic pressure in a bioreactor was implemented to promote cell ingrowth.

In order to fulfill their role in cardiovascular tissue engineering, seeded cells, together with newly recruited host cells, should differentiate to endothelial cells with anti-thrombotic properties and medial smooth muscle cells that ensure the contractility and production of extracellular matrix [62]. Allogeneic WJCs represent an advantageous cell source since they require no autologous harvesting procedure and can differentiate in vitro into endothelial cells [42] and smooth muscle cells [43,63]. In our study, the formation of neo-intima containing alpha-smooth muscle actin-positive cells and the formation of a CD31-positive endothelial lining most likely occurred through trans-anastomotic overgrowth (Figure 14). This took place regardless of seeding with ASCs or WJCs since the formation and endothelialization of neo-intima had similar patterns in all patches (Figure 9). Yet, WJCs seeded on decellularized pericardia reduced the histological grade of NAI and patch resorption to a greater degree than the ASC-seeded matrix. Recellularization with WJCs also mitigated NIH on the xenograft patch. Given the fact that seeded grafts transform into neo-arteries via an inflammation-mediated process [33], we assume that these histological changes demonstrated the immunomodulatory effects of WJCs on the remodeling of tissue-engineered patches. Optimally, seeded biological scaffolds are gradually replaced with host tissue while maintaining structural integrity. Rapid matrix resorption or an excessive inflammatory reaction may result in aneurysm formation or graft failure [64,65]. Hypothetically, slowing down patch resorption may become beneficial for the stabilization of newly formed vascular tissue. Taken together, our in vivo results confirm that WJCs are suitable candidates for vascular tissue engineering. 

### 3.3. Clinical Context and Implications

Arterial conduits, used in cardiac bypass surgery, have better patency rates than do vein grafts. The use of saphenous vein grafts in coronary and peripheral arterial surgery is associated with harvest injury, endothelial damage, constrictive inward remodeling, and intimal hyperplasia, all of which potentially lead to vein graft failure [66]. Intimal hyperplasia develops as a response to vascular injury in native vessels, stents, and autologous grafts; it also develops in anastomoses of vascular prostheses due to a lack of endothelium as well as to mismatches in diameter and compliance [4]. These clinical phenomena were somewhat observed in our animal experiment. The arterial autograft highly resembled the native artery, while the venous autograft developed NAI and NIH according to a histological examination.

Bovine pericardium (BP) has become popular in clinical cardiovascular surgery. When compared with primary closure of the arteriotomy, utilizing a patch angioplasty during a traditional carotid endarterectomy generally has a more favorable effect in reducing the combined perioperative and long-term risks of stroke, perioperative arterial occlusion, and restenosis [67]. A recent meta-analysis of randomized trials comparing the effectiveness of BP to that of venous and synthetic patch materials in the closure of a carotid endarterectomy incision presented equivalent clinical outcomes [68]. However, BP was more favorable than PET with respect to surgical handling, reduced suture line bleeding, and potential use in infected fields [69], such as in endocarditis [70,71] or vascular graft infection [13,72]. A single-center study focusing on endarterectomies of the common femoral artery revealed no association between patch type (venous, bovine, or prosthetic) and the occurrence of surgical site infection in the groin [73]. Concerning cardiac surgery, there were no significant differences between PTFE and BP in outcomes after the closure of cardiac ventricular septal defects. However, BP was preferred because of its ease of surgical handling, elasticity, and associated lower risk of endocarditis [74]. 

As for human clinical application of cell-seeded patches, Shin’oka et al. [75] implanted 19 tissue-engineered patches seeded with bone marrow cells into the pulmonary arteries of pediatric patients with congenital heart anomalies. There were no graft-related complications. These patches, however, were composed of synthetic degradable copolymers as opposed to decellularized tissue. Clinical use of cell-seeded decellularized vascular matrices has rarely been reported. Published reports include an autologous cell-seeded decellularized vein homograft to replace the iliac vein in one case [27] and a portal vein bypass procedure in three cases [28,29]. There are still concerns about the degradation and disintegration of decellularized arterial implants [65]. We are unaware of any clinical implantation of the seeded pericardia to date.

We recognize that direct application of our experimental results to clinical medicine is not yet feasible. An ideal cardiovascular biomaterial should be off-the-shelf and available in various sizes. It should be biocompatible and resistant to infection, rejection, and thrombosis. Moreover, the material should permit growth and remodeling in conjunction with host cells [76]. Seeded pericardial patches are labor-intensive and costly; additionally, they require a prolonged fabrication time and storage in a sterile environment. Theoretically, they could be cryopreserved to provide immediate availability. However, current experience with the cryopreservation of living, seeded grafts is limited [77,78] and this technology requires further research. Despite these limitations, the seeded pericardial patch, with its biocompatibility and potential for growth and remodeling, is becoming increasingly resemblant of an ideal graft.

## 4. Materials and Methods

### 4.1. Sample Preparation

#### 4.1.1. Control Patches

An autologous arterial graft was created by retrieving one of the two carotid arteries. Pigs tolerate carotid resection or occlusion well. They do not develop symptoms of cerebral ischemia. This autograft was longitudinally cut open to create a planar patch that was implanted into the contralateral carotid. An autologous vein graft was created by retrieving an internal jugular vein that runs close to the carotid artery. This vein autograft was longitudinally cut open to form a planar patch that was implanted into the ipsilateral carotid artery. Retrieval of the jugular vein is also well tolerated by pigs.

The PET Vascutek^®^ graft was purchased from Vascutek Ltd., a Terumo Company, Renfrewshire, Scotland. It is a thin-walled gelatin-impregnated knitted carotid patch with a fluoropolymer surface [79]. The ePTFE Propaten^®^ Gore^®^ graft was purchased from W. L. Gore & Associates, Inc., Flagstaff, AZ, USA. This heparin-coated thin-walled stretch vascular graft is configured for a pediatric shunt [80]. A tubular graft with an internal diameter of 5 mm was longitudinally cut open and adjusted to form a patch.

The XenoSure^®^ biological patch was purchased from LeMaitre Vascular, Inc., Richmond, BC, Canada. It is a bovine pericardium chemically treated with glutaraldehyde. The material is intended for use as surgical patch material for cardiac and vascular reconstruction and repair, soft tissue deficiency repair, and reinforcing the suture line during general surgical procedures [81]. The BioIntegral Surgical^®^ No-React^®^ biological patch was purchased from MAC Medical, BioIntegral Surgical Inc., Mississauga, ON, Canada. It was a porcine pericardium treated with proprietary detoxification of a glutaraldehyde-treated tissue. The detoxified material should not leach GA molecules and is intended for use as an intracardiac patch to close intercavity defects and enlarge the aortic root and in carotid endarterectomies and pericardial closures [54].

#### 4.1.2. Experimental Patches

We fabricated the experimental pericardial patches as previously described [82]. Briefly, the porcine and ovine pericardia (obtained from other terminated experiments) were decellularized in an automated decellularization system using sodium dodecyl sulfate (SDS), DNAse, and deionized water. The tissues were then sterilized using an ethanol solution and analyzed using DAPI-stained thin sections (see Figure 2). These decellularized matrices were reseeded with porcine autologous ASCs or allogenous WJCs. The harvesting and characterization of ASCs were previously described [82].

We used new WJCs, which were isolated from umbilical cord tissues (UCTs) collected from newborn piglets on the experimental farm of the Institute of Animal Science. Immediately after collection, UCTs were lavaged using 200 μg/mL of gentamicin in saline and placed under sterile conditions. After the blood vessels were removed, the remaining tissue was chopped into small pieces (2–5 mm^3^). The UCT explants were then centrifuged, washed, resuspended in a growth medium (DMEM medium supplemented with 15% porcine serum and 20 μg/mL of gentamicin), placed in Petri dishes, and incubated at 37 °C in an atmosphere with saturated humidity and CO_2_ levels of 5%. WJCs that migrated from UCT explants were cultured up to passage 2 and then evaluated through viability and flow cytometry analysis. Isolated WJCs were also analyzed using a NovoCyte Flow Cytometer (Acea Biosciences, Inc., San Diego, CA, USA). Flow cytometry results for WJCs are presented in Table 2.

**Figure 15:**
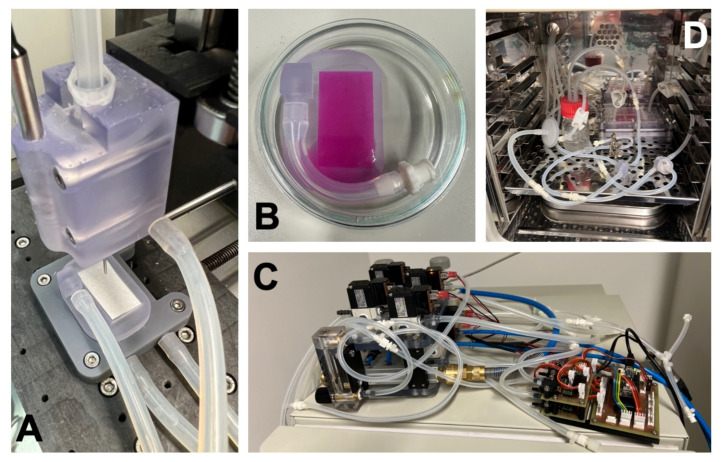
(**A**) A vacuum fixture with a decellularized pericardium installed in a bioprinter bed. (**B**) Upon completion of printing, the reservoir of the fixture is filled with a culture medium and placed into a Petri dish for the initial adhesion phase. (**C**) The stimulation and perfusion system. (**D**) Cell-seeded decellularized tissue installed in a bioreactor chamber for dynamic cultivation and stimulation.

The seeding of cells on decellularized matrices was carried out using a modified 3D bioprinter. Decellularized tissues were fixed in a custom-built vacuum holder in a bioprinter printbed. The cell suspension (density: 10 million cells/mL) was then printed onto the surface with an optimized toolpath, ensuring a homogenous spread of cells with a resulting density of 90,000 cells/cm^2^. Once the printing process was complete, the samples were placed in an incubator for 60 min in order to allow the cells to adhere to the surface. The matrices were fixed in the chamber of the bioreactor and cultivated for 5 days. The printing and fixing processes, which took place in the bioreactor chamber, are illustrated in Figure 15. Stimulation was set as described in [82], i.e., 5.9/10.6 kPa (120/80 mmHg) high/low pressure with a frequency of 1 Hz (60 pulses per minute) and cycled with mild perfusion at a flow rate of 2 mL/min. This dynamic cultivation promoted cell ingrowth into decellularized tissue and differentiation of ASCs into smooth muscle cells [82].

### 4.2. Surgical Experiment In Vivo

#### 4.2.1. Adipose Tissue Harvest

We used healthy juvenile female domestic Prestice black pied pigs (sus scrofa domesticus). The animals were given 2 weeks for accommodation. The pigs were given no food 24 h prior to general anesthesia (GAn), which was achieved through routine means. Premedication involved intramuscular (i.m.) administration of ketamin, azaperon, and atropin. GAn was induced using intravenous (i.v.) propofol. The animals were intubated, and anesthesia was maintained through the inhalation of isofluran (1.5%), oxygen (0.5 L/min), and air (0.5 L/min). Analgesia was provided in the form of 2.5–4 µg/kg/h fentanyl (i.v.), and postoperative analgesia was achieved through the administration of transcutaneous fentanyl (Transtec 35 µg/h). The pigs were housed under standard husbandry conditions and were given food and water ad libitum. The opportunity for social interaction was assured. Subcutaneous adipose tissue was retrieved (under GAn) for ASC isolation through a small incision in the neck region and later used for implantation in the form of ASC-recellularized patches. The pigs had a mean body weight of 32 ± 2 kg at the time of fat tissue retrieval.

#### 4.2.2. Implantation

Under GAn, both carotid arteries were surgically exposed with two incisions in the neck region. Heparin (200 IU/kg) was administered 2 min prior to cross-clamping of the vessel, and redosing was implemented according to the activated clotting time in order to ensure full intraoperative anticoagulation. Once the carotid artery was cross clamped, a 2–3-cm-long longitudinal arteriotomy was performed. The artery was then flushed with heparinized saline. The patch graft was implanted using a running 7/0 polypropylene suture. The artery was unclamped and bleeding was controlled. A heparin reversal agent was not required. The boundaries of the implanted patch were marked with metal clips. The wound was sutured in anatomical layers. Prophylactic single-dose antibiotics were administered intramuscularly (amoxicillin, Betamox). Acetylsalicylic acid was administered as a thromboprophylaxis drug at a dosage of 100 mg once daily. The pigs had a mean body weight of 41 ± 9 kg at implantation.

For the pericardial patches, we did not distinguish between serous and fibrous sides of the pericardium. Moreover, the seeded cells were evenly distributed throughout the walls of the recellularized patches. Some studies involving pericardium implantation into a vessel mention and address the front and reverse sides of the pericardial matrix [50,55], while others do not [56,57]. Implantation experiments were carried out in duplicates.

#### 4.2.3. Flowmetry

Blood flow volume was measured with a flow probe (Transonic, ADInstruments) on the surgically exposed native artery immediately after patch implantation and just prior to explantation. The muscular and elastic porcine carotid artery is prone to spasms caused by surgical dissection and vessel clamping. In certain cases, we were somewhat successful in inducing relaxation with a local application of papaverine. However, flowmetry may have provided us with frequent false-low results. The highest flow rate was taken into account.

#### 4.2.4. Angiography

Immediately prior to surgical dissection and explantation, we carried out an angiography (under GAn) with an iodinated contrast agent via femoral access in order to evaluate vessel patency or potential stenosis. We implemented the Seldinger technique, a mobile C-arm, and a translucent operating table. Unlike humans, the common carotid arteries in pigs emerge from a common carotid trunk that originates from the brachiocephalic trunk and aortic arch [83]. We achieved selective catheterization of the carotid arteries through the bi-carotid trunk. Digital subtraction angiograms and skiagrams were obtained in two projections (anteroposterior and lateral) in order to assess vessel morphology thoroughly. Metal clips indicated implant location.

#### 4.2.5. Explantation

After 1 month (i.e., 28 days), the constructs were carefully explanted under GAn. The pigs had a mean body weight of 57 ± 8 kg at explantation. The carotid artery was surgically dissected from postoperative adhesions and the distal pulse was evaluated. We did not administer heparin during explantation. After cross-clamping, the artery was quickly excised, gently flushed with warm saline, and cut into cross-sections. A gross examination of the flow surface of the explants was carried out to check for patency, pannus formation, or the presence of a potential thrombus. The animals were euthanized under GAn with a thiopental and potassium chloride overdose.

### 4.3. Microscopical Examination

#### 4.3.1. Histology and Immunohistochemistry

After explantation, the constructs were cross-sectioned and fixed with 4% formaldehyde and embedded in paraffin. Midgraft sections were mounted on glass, dried, and stained with hematoxylin and eosin (Merck & Co., Inc., Kenilworth, NJ, USA) and Van Gieson Elastica (proprietary method) to assess tissue microarchitecture. We examined the immunohistochemical expression of alpha-smooth muscle actin (primary antibody: Cell Marque Corp., Rocklin, CA, USA; secondary antibody: Ventana, Roche Group, Tucson, AZ, USA) and the endothelial marker CD31 (primary antibody: Novus Biologicals, LLC, Centennial, CO, USA; secondary antibody: Vector Laboratories, Inc., Burlingame, CA, USA). The implanted pericardial patch was a non-vascular tissue without a typical arrangement comprising three main layers: intima, media, and adventitia. We termed newly formed luminal tissue and ab-luminal tissue neo-intima and neo-adventitia, respectively. 

#### 4.3.2. Neo-Intimal Hyperplasia Thickness

We used QuPath open-source software (version 0.3.0) for digital pathology image analysis [84]. The thickness of the neo-intimal hyperplasia underneath the patches was measured in three to six points in radial directions. Because of the possibly different diameters of vessels and sizes of patches between individual animals, the absolute values of NIH thicknesses cannot be directly compared between the used implants. For this reason, and in order to minimize the effect of potential tissue shrinkage during processing and to facilitate comparison between samples, we divided the mean thickness of the NIH by the mean thickness of the native vessel wall opposite the implanted patch. Using this relative NIH, a value of 1 would mean that the thickness of the implant NIH is equal to that of the vessel wall. We further chose ePTFE as a baseline material, and we normalized the relative NIH thickness of other surfaces to that of ePTFE.

#### 4.3.3. Grading of Neo-Adventitial Inflammation, Patch Resorption, Calcifications, and Neo-Intimal Hyperplasia

We classified the histological signs of neo-adventitial inflammation, patch resorption, calcifications, and neo-intimal hyperplasia into four grades: 0—minimal or none; 1—mild; 2—moderate; and 3—advanced. 

### 4.4. Hematology

Peripheral blood was sampled at implantation and explantation in order to examine microscopic differential blood counts. Elevated white blood cell levels would non-specifically indicate the presence of immune activation.

### 4.5. Statistics

Data with a normal distribution are expressed as the mean and standard deviation (SD) and were computed in a Microsoft^®^ Excel spreadsheet. Data sets were compared by a two-tailed Student’s *t*-test. A *p*-value less than 0.05 (≤0.05) was considered significant. 

The statistical significance of NIH values was evaluated using nonparametric Kruskal–Wallis One Way Analysis of Variance on Ranks, Dunn’s Method, and statistical significance (*p* ≤ 0.05) in MATLAB. Due to the limited number of experimental animals in this study, some conclusions are stated based on trend values obtained.

## 5. Conclusions

In the present study, we developed tissue-engineered vascular patches through the decellularization of pericardial matrices and recellularization with autologous adipose tissue-derived stromal cells or allogeneic Wharton’s jelly mesenchymal stem cells. In a model of vascular patch implantation in a porcine carotid artery, all experimental pericardial decellularized and recellularized patches, as well as all control patches, were well-integrated and patent with no signs of thrombosis, stenosis, or aneurysm. 

A histological analysis revealed the presence of an endothelial lining. The arterial autograft resembled a native artery. All other control and experimental patches developed neo-intimal hyperplasia and neo-adventitial inflammation, which were most prominent in XenoSure^®^ and Xeno-decel and least prominent in NoReact^®^. In xenografts, the degree of neo-intimal hyperplasia developed in the following order: Xeno-decel > Xeno-ASC > Xeno-WJC. Neo-adventitial inflammation and patch resorption increased in Allo-ASC and Xeno-ASC and decreased in Allo-WJC and Xeno-WJC. 

In our setting, pre-implant seeding with ASCs or WJCs had a modest impact on vascular patch remodeling. However, these changes indicate that ASCs elicited accelerated remodeling and WJCs evoked immunomodulatory properties. 

## Figures and Tables

**Table 1:** A list of the control and experimental pericardial decellularized and recellularized patches.

Control Patches					
Autografts		Prosthetic		Bioprosthetic pericardial	
Arterial	Venous	ePTFE Gore^®^	PET Vascutek^®^	XenoSure^®^	NoReact^®^
**Experimental Pericardial Patches**					
Porcine allografts			Ovine xenografts		
Allo-Decel	Allo-ASC	Allo-WJC	Xeno-Decel	Xeno-ASC	Xeno-WJC

Abbreviations: Allo, allogeneic; WJC, allogeneic Wharton’s jelly mesenchymal stem cell; ASC, autologous adipose tissue-derived stromal cell; decel, decellularized; ePFTE, expanded polytetrafluoroethylene; PET, polyethylene terephthalate; Xeno, xenogeneic.

**Table 2:** Flow cytometry analysis of isolated porcine Wharton´s jelly mesenchymal stem cells (pWJCs).

CD Marker	pWJC	Antibody Provider	Cat. No.
29	99.56%	Invitrogen (Waltham, CA, USA)	CD2920
31	0.26%	Origene (Rockville, MD, USA)	SM2146APC
34	0.38%	Biorbyt (Cambridge, UK)	orb247244
45	0.70%	Biorad (Hercules, CA, USA)	MCA1222A647
90	99.34%	BD Biosciences (Franklin Lakes, NJ, USA)	555596
105	86.35%	Abcam (Bristol, UK)	ab53321
146	8.43%	Origene (Rockville, MD, USA)	SM1860F

## Data Availability

Not applicable.

## Supplementary Materials

The following supporting information can be downloaded at: www.mdpi.com/article/10.3390/ijms23063310/s1.
