# Supplementary material for: Vascular Remodeling of Clinically Used Patches and Decellularized Pericardial Matrices Recellularized with Autologous or Allogeneic Cells in a Porcine Carotid Artery Model
Source: Int J Mol Sci. 2022 Mar 18;23(6):3310. doi: 10.3390/ijms23063310 (PMC8954945; doi:10.3390/ijms23063310)
Supplement: Supplementary file 1 [file ijms-23-03310-s001.zip › Supplementary material IJMS 1601168_new with Suppl Fig 13.pdf]

Supplementary material IJMS 1601168

Control patches

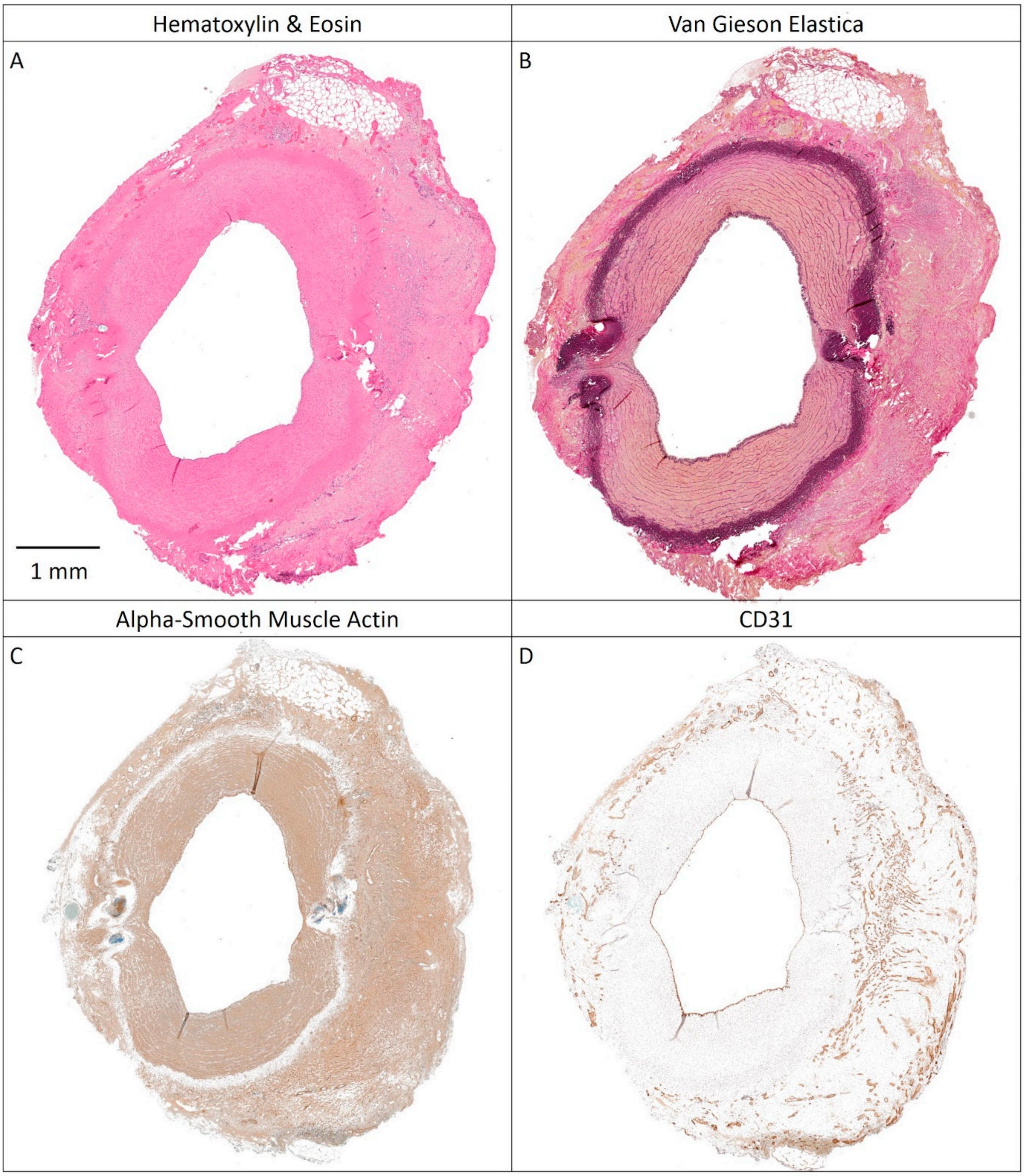

Supplementary Figure S1. Arterial autografts - histology and immunohistochemistry of vascular patches in porcine carotid arteries 1 month post-implantation. Representative cross-sections of the midgraft regions are shown. Implanted patches comprise the upper half of the vessel, magnification 20×.

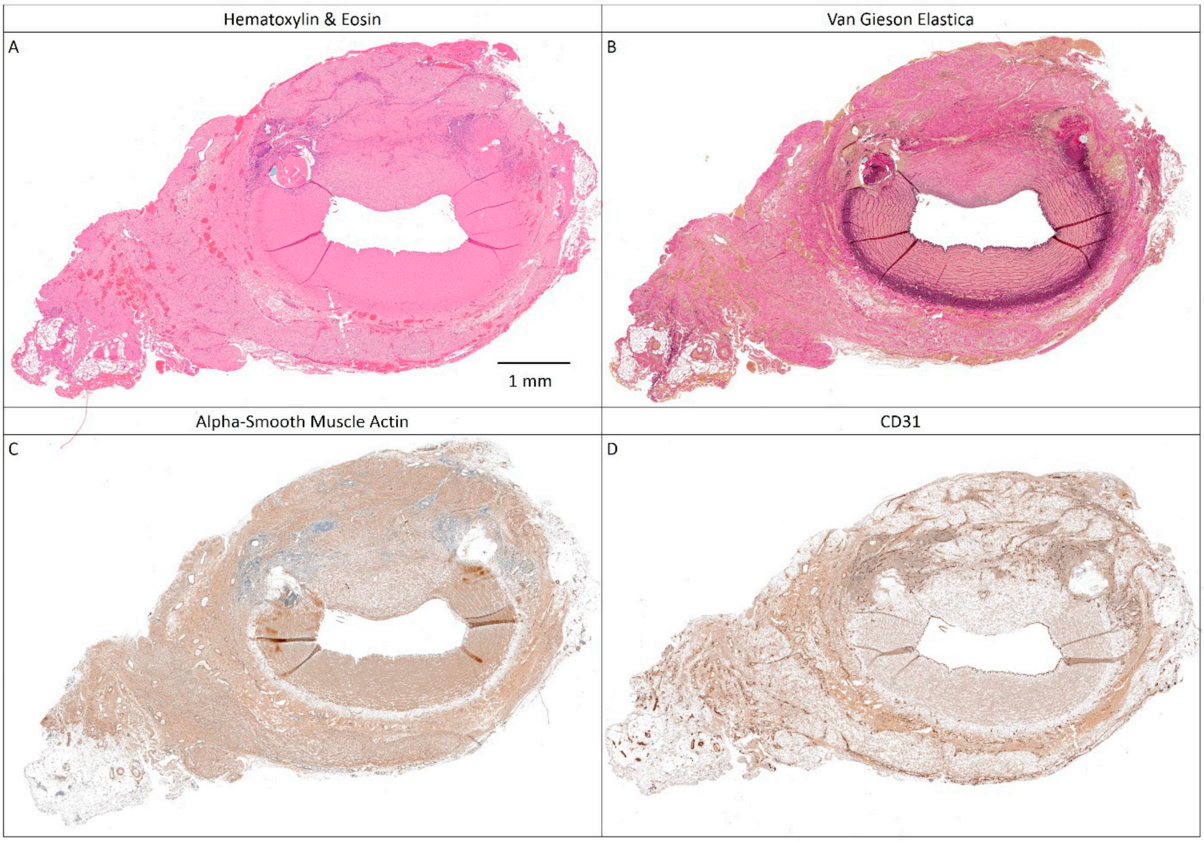

Supplementary Figure S2. Venous autografts - histology and immunohistochemistry of vascular patches in porcine carotid arteries 1 month post-implantation. Representative cross-sections of the midgraft regions are shown. Implanted patches comprise the upper half of the vessel, magnification 20×.

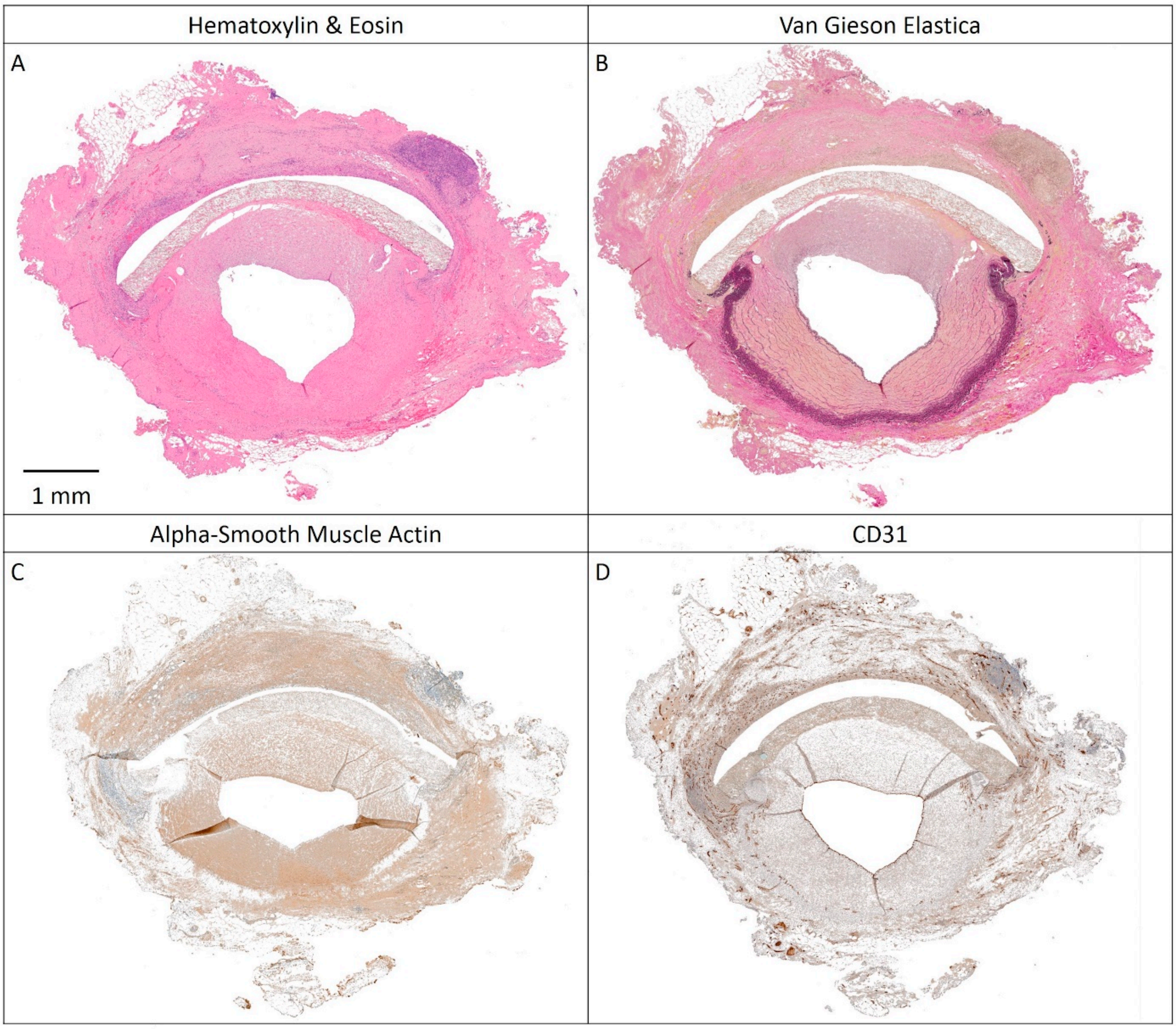

Supplementary Figure S3. Expanded polytetrafluoroethylene (ePTFE Propaten® Gore®) - histology and immunohistochemistry of vascular patches in porcine carotid arteries 1 month post-implantation. Representative cross-sections of the midgraft regions are shown. Implanted patches comprise the upper half of the vessel, magnification 20×.

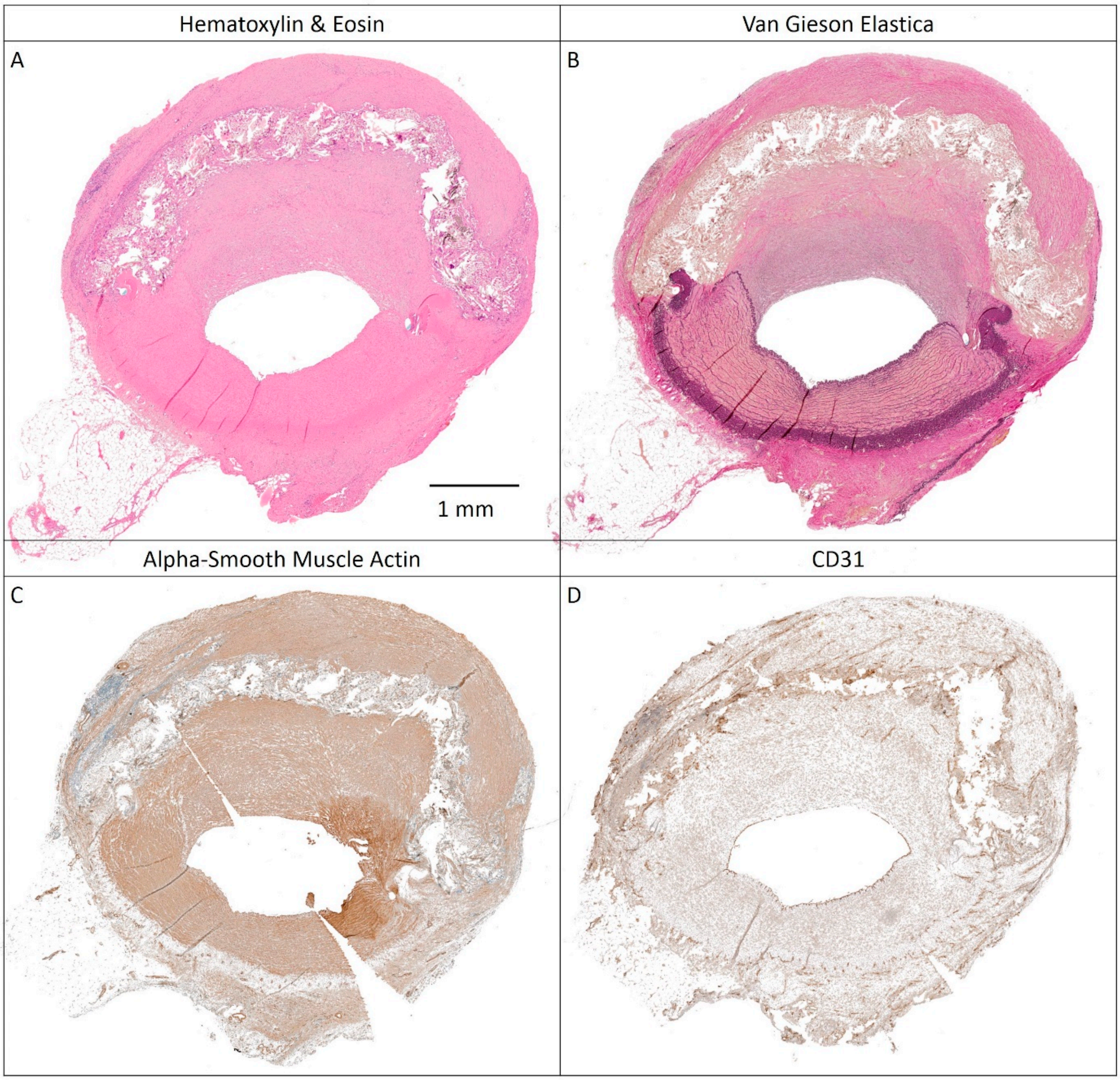

Supplementary Figure S4. Polyethylene terephthalate (PET Vascutek®) - histology and immunohistochemistry of vascular patches in porcine carotid arteries 1 month post-implantation. Representative cross-sections of the midgraft regions are shown. Implanted patches comprise the upper half of the vessel, magnification 20×.

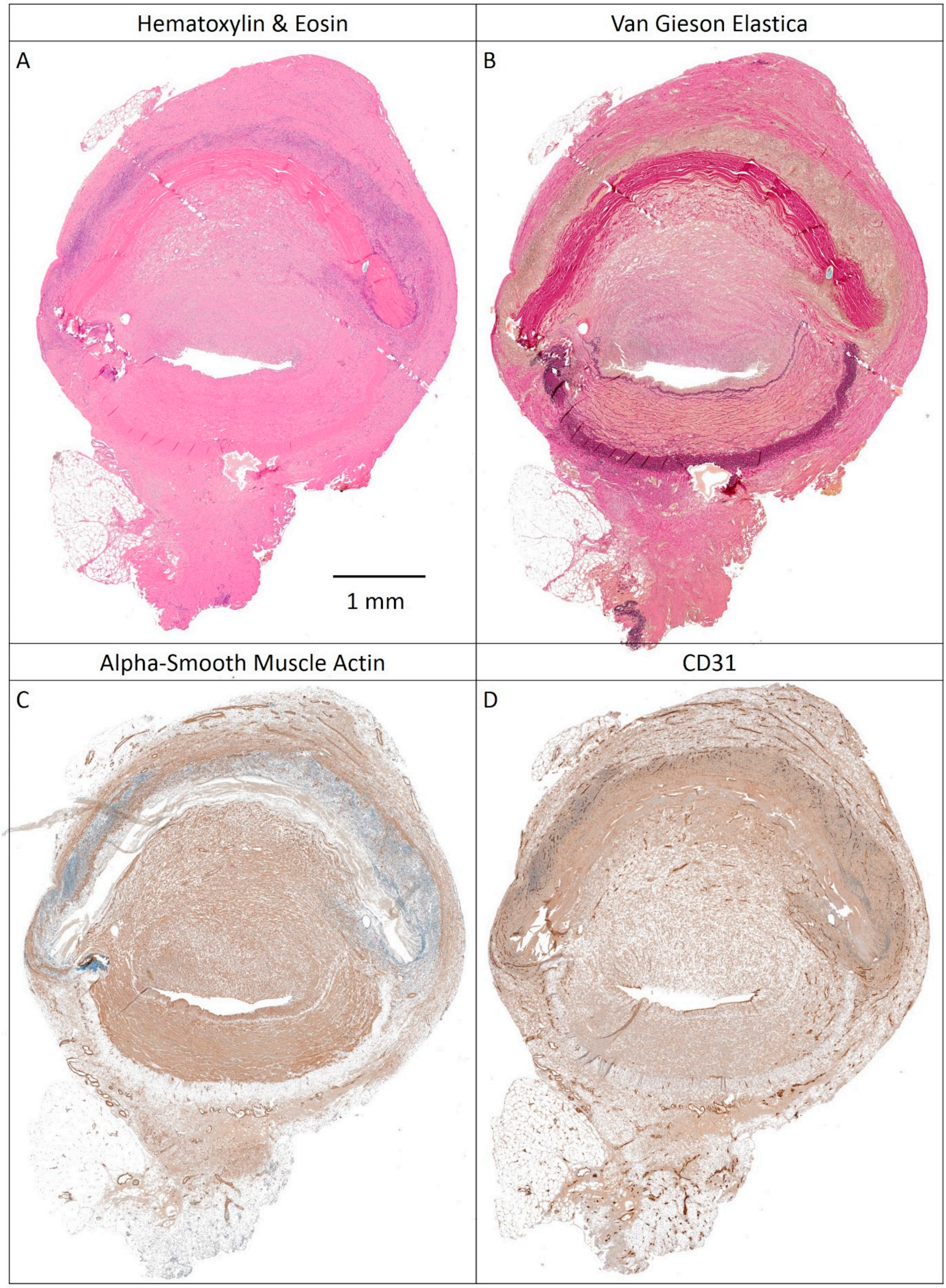

Supplementary Figure S5. Bovine pericardium fixed with glutaraldehyde (XenoSure®) - histology and immunohistochemistry of vascular patches in porcine carotid arteries 1 month post-implantation. Representative cross-sections of the midgraft regions are shown. Implanted patches comprise the upper half of the vessel, magnification 20×.

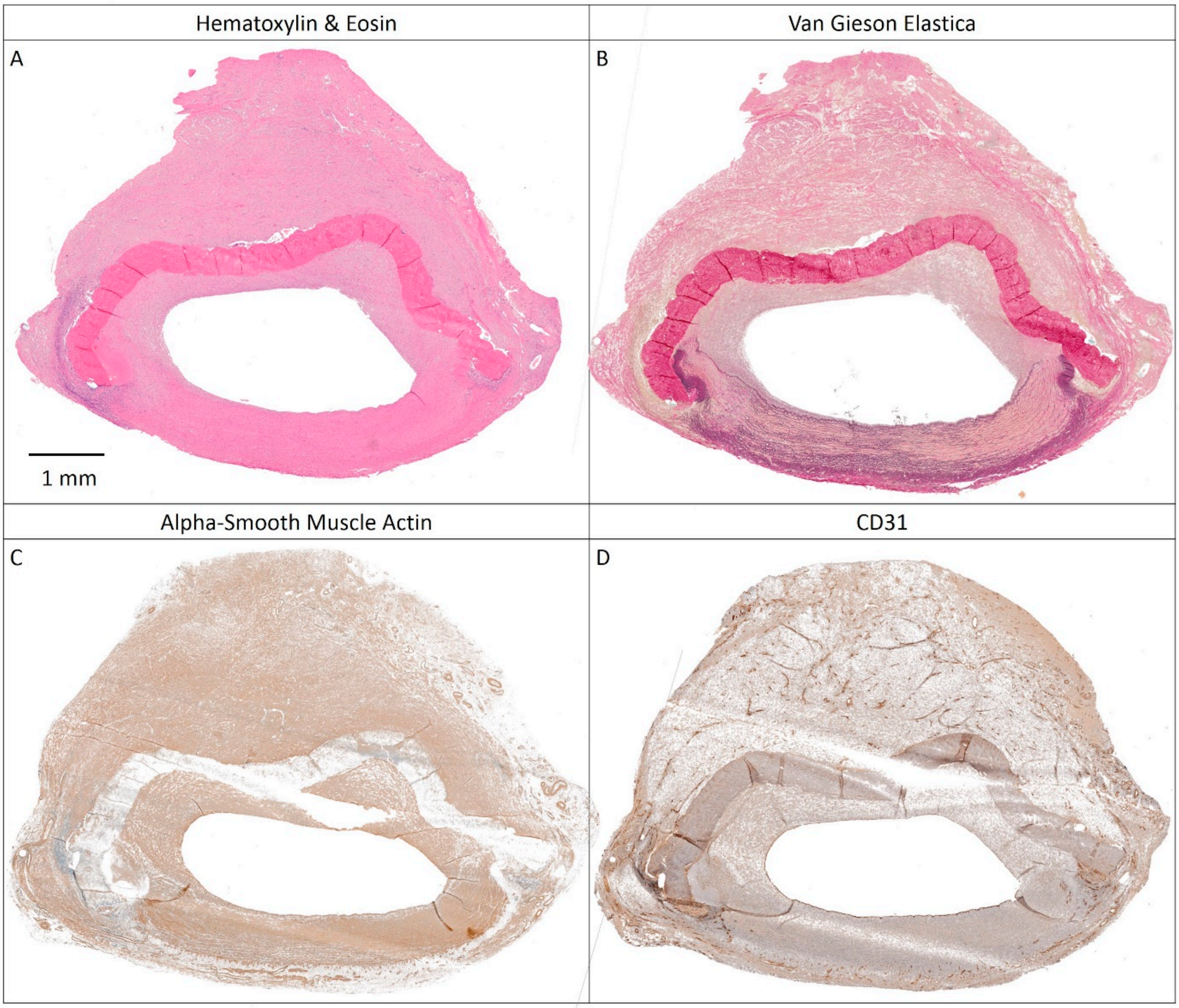

Supplementary Figure S6. Detoxified porcine pericardium (NoReact®) - histology and immunohistochemistry of vascular patches in porcine carotid arteries 1 month post-implantation. Representative cross-sections of the midgraft regions are shown. Implanted patches comprise the upper half of the vessel, magnification 20×.

Experimental pericardial patches

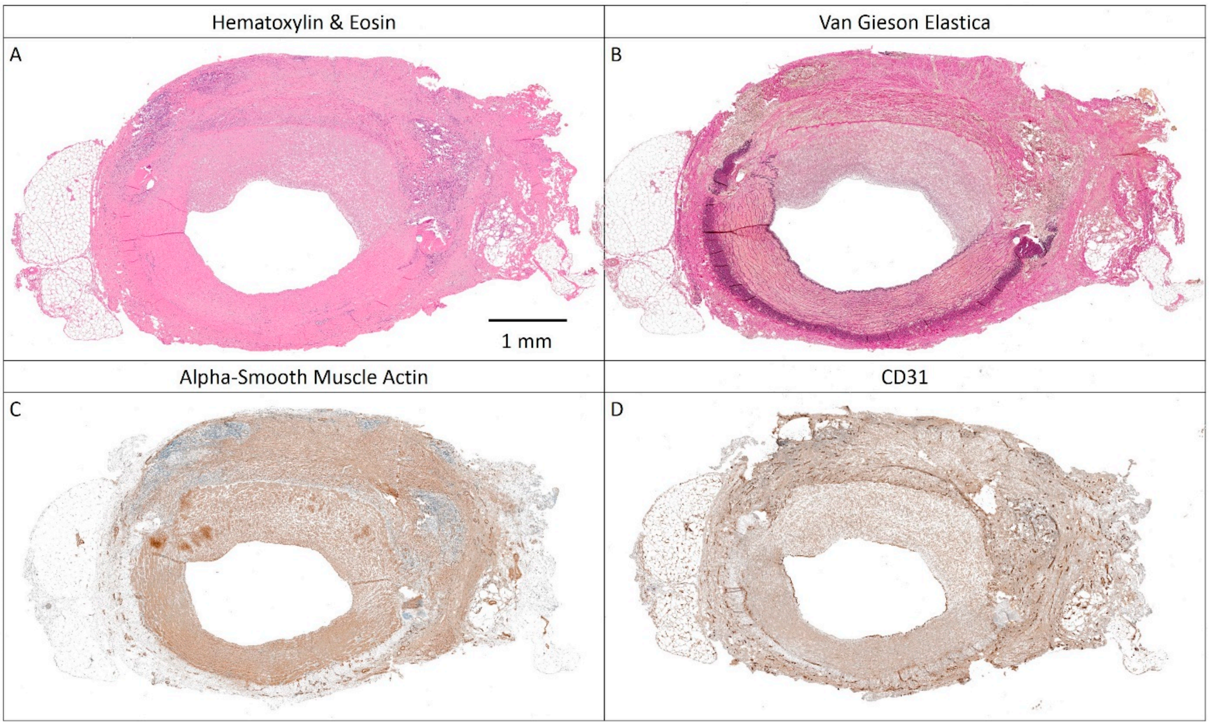

Supplementary Figure S7. Decellularized porcine allograft pericardium (Allo Decel) - histology and immunohistochemistry of vascular patches in porcine carotid arteries 1 month post-implantation. Representative cross-sections of the midgraft regions are shown. Implanted patches comprise the upper half of the vessel, magnification 20×.

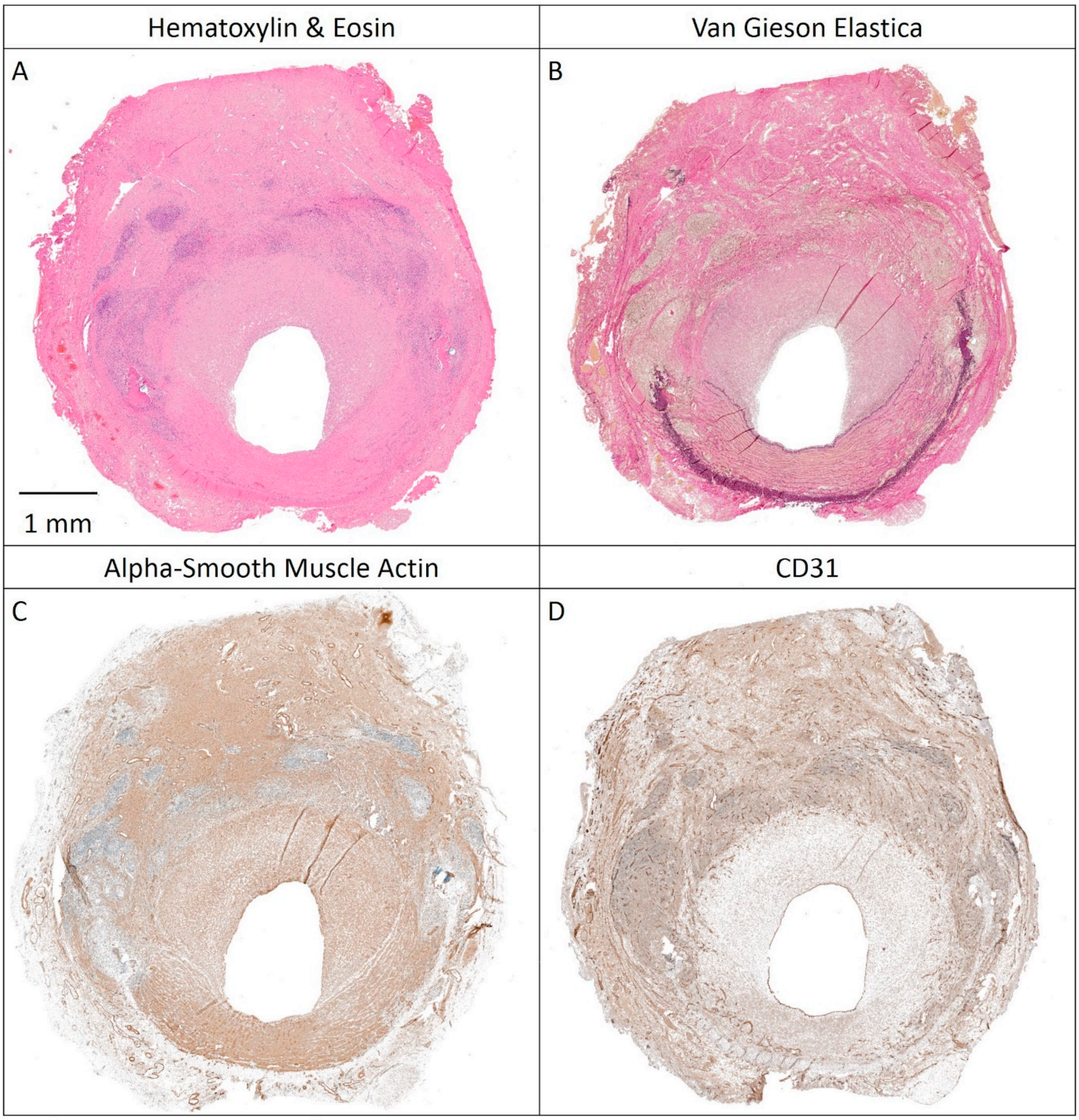

Supplementary Figure S8. Decellularized porcine allograft pericardium recellularized recellularized with autologous adipose tissue-derived stromal cells (Allo ASC) - histology and immunohistochemistry of vascular patches in porcine carotid arteries 1 month post-implantation. Representative cross-sections of the midgraft regions are shown. Implanted patches comprise the upper half of the vessel, magnification 20×.

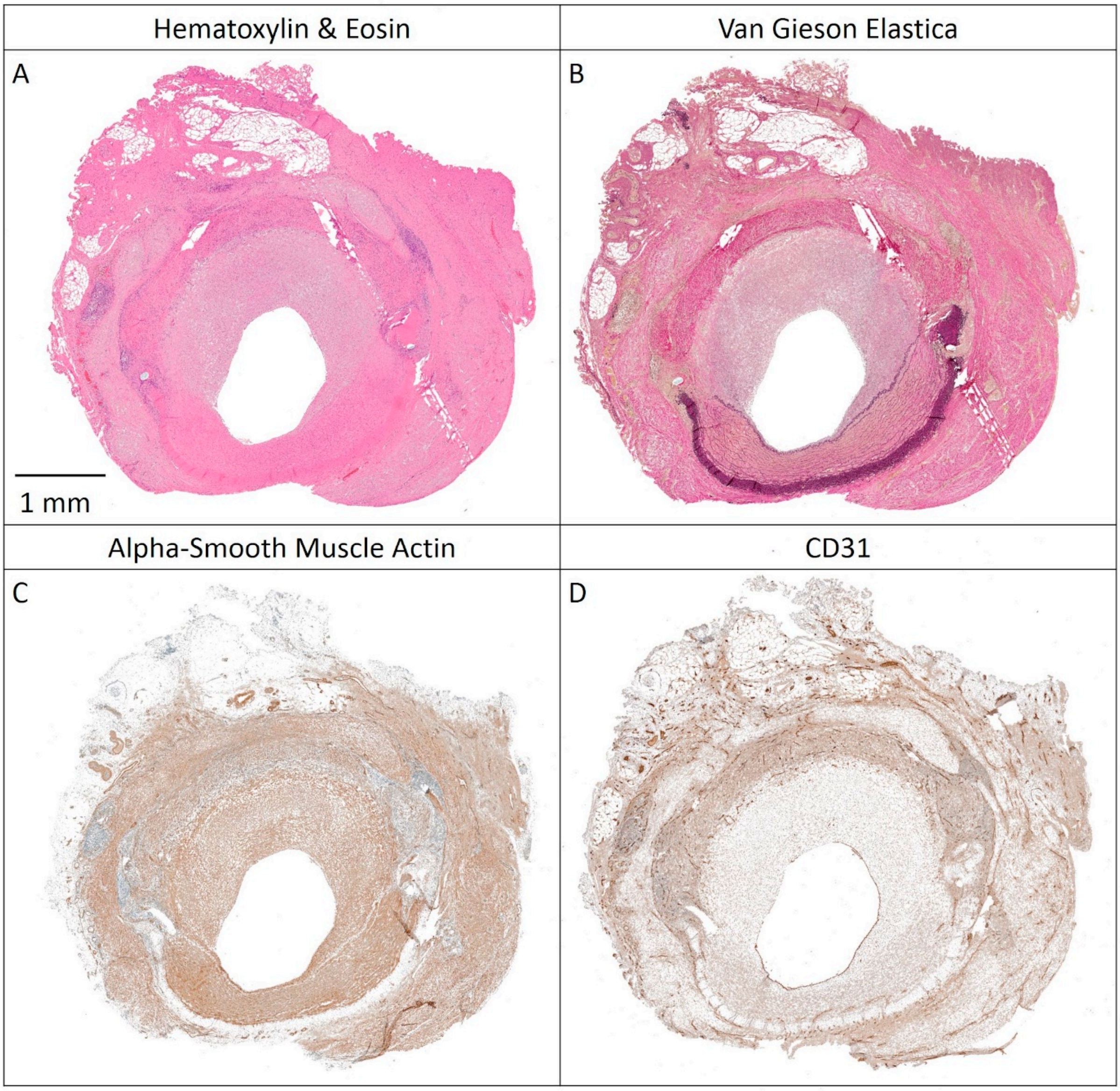

Supplementary Figure S9. Decellularized porcine allograft pericardium recellularized with allogeneic Wharton's jelly mesenchymal stem cells (Allo WJC) - histology and immunohistochemistry of vascular patches in porcine carotid arteries 1 month post-implantation. Representative cross-sections of the midgraft regions are shown. Implanted patches comprise the upper half of the vessel, magnification 20×.

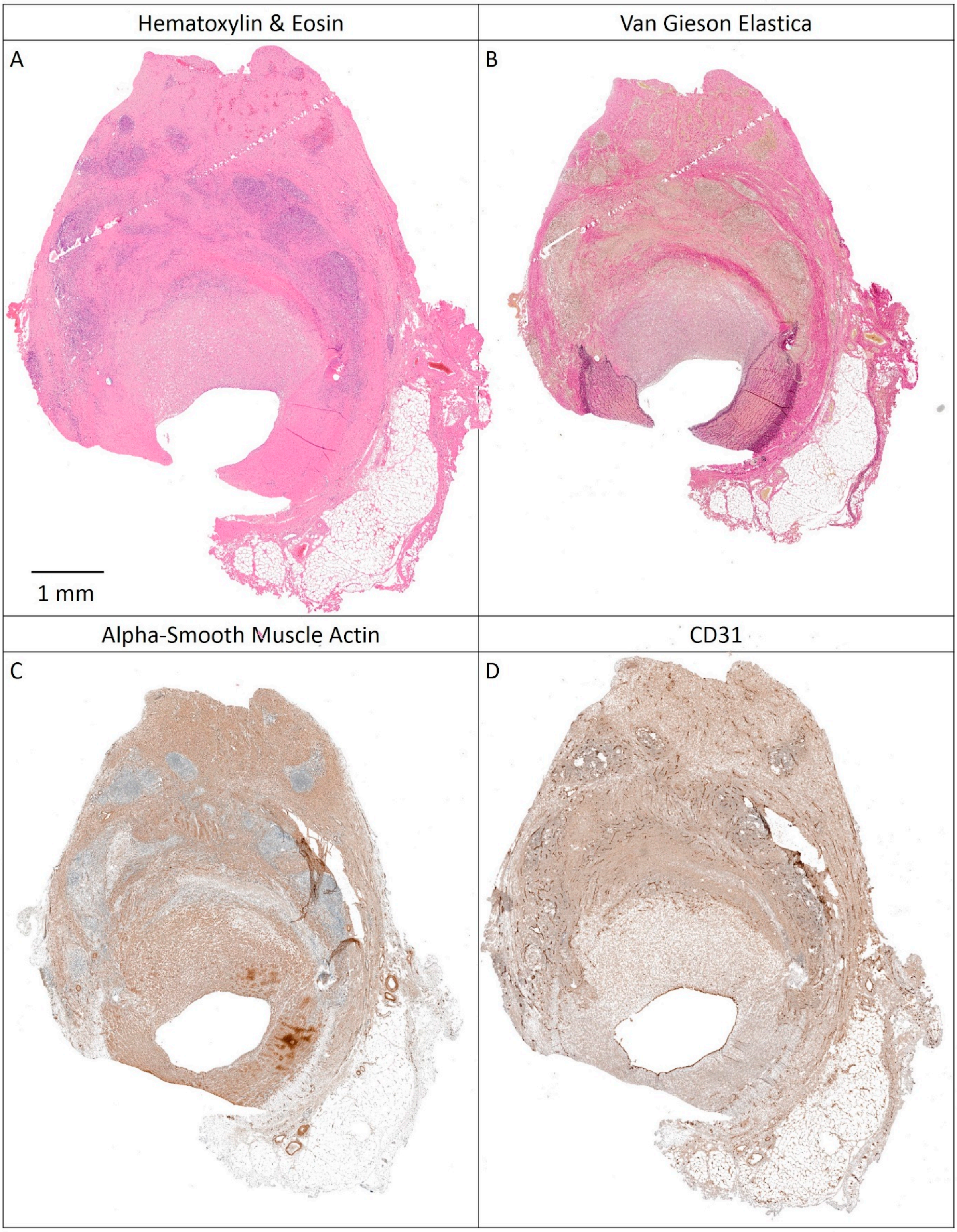

Supplementary Figure S10. Decellularized ovine xenograft pericardium (Xeno Decel) - histology and immunohistochemistry of vascular patches in porcine carotid arteries 1 month post-implantation. Representative cross-sections of the midgraft regions are shown. Implanted patches comprise the upper half of the vessel, magnification 20×.

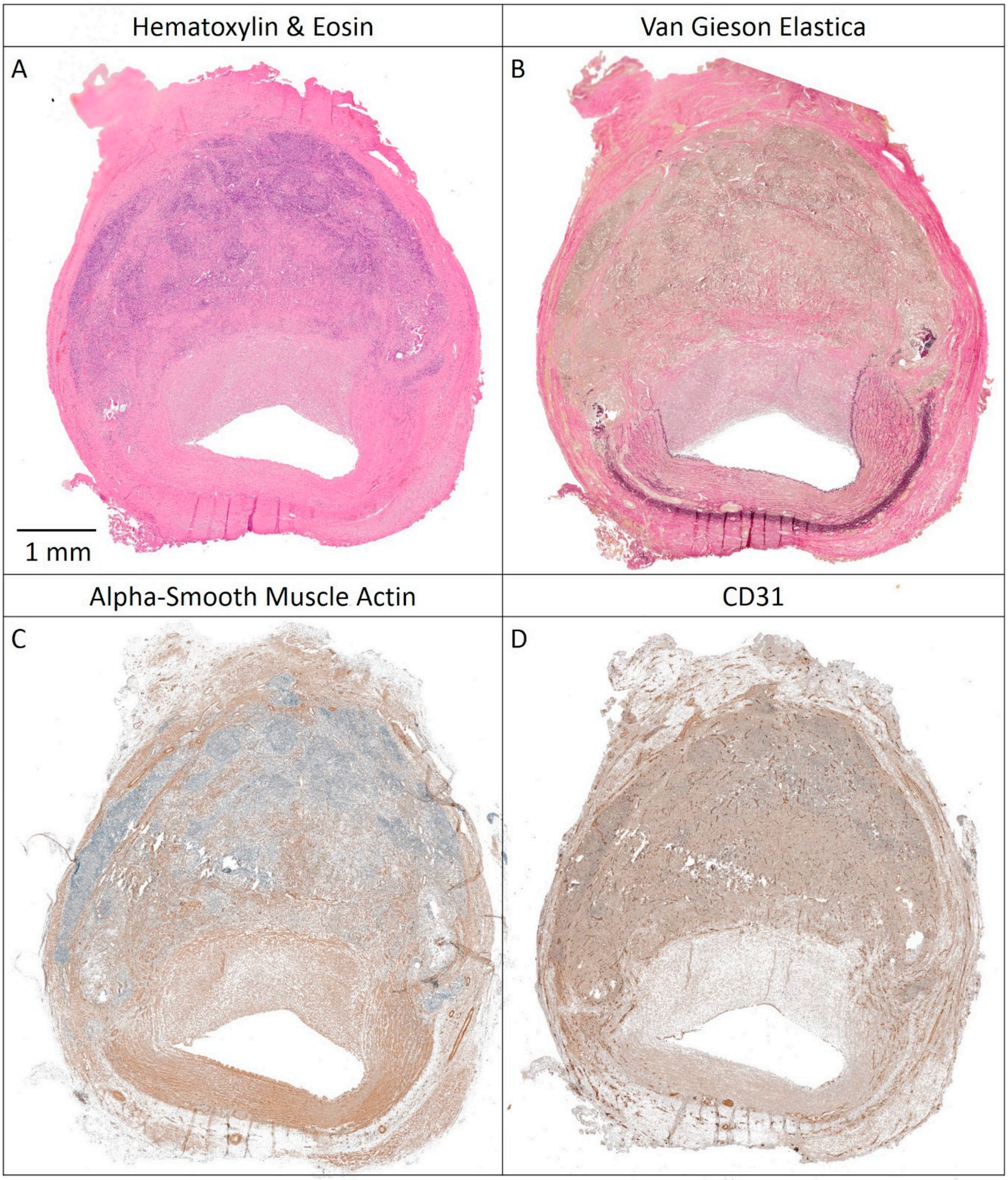

Supplementary Figure S11. Decellularized ovine xenograft pericardium recellularized with autologous adipose tissue-derived stromal cells (Xeno ASC) - histology and immunohistochemistry of vascular patches in porcine carotid arteries 1 month post-implantation. Representative cross-sections of the midgraft regions are shown. Implanted patches comprise the upper half of the vessel, magnification 20×.

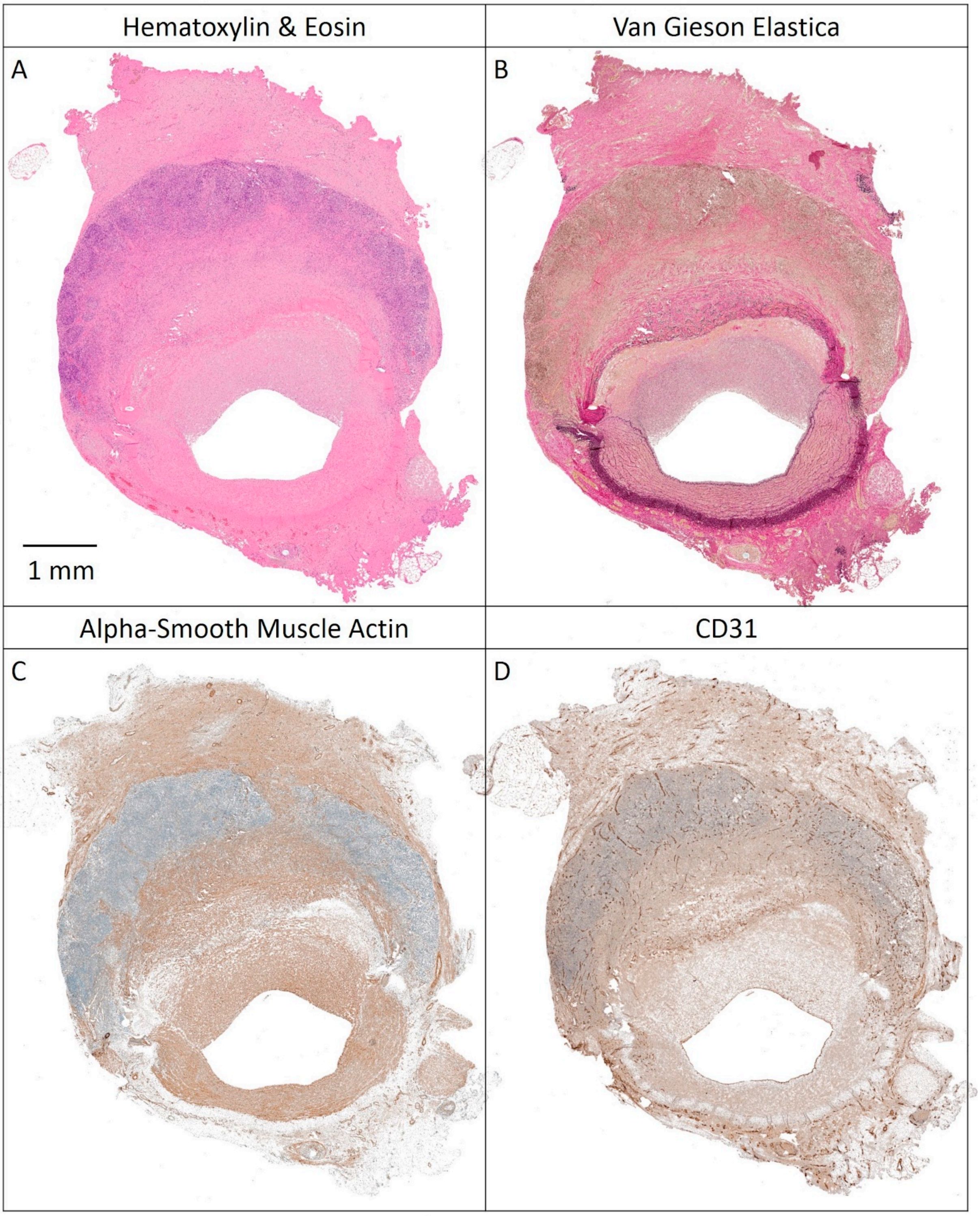

Supplementary Figure S12. Decellularized ovine xenograft pericardium recellularized with allogeneic Wharton's jelly mesenchymal stem cells (Xeno WJC) - histology and immunohistochemistry of vascular patches in porcine carotid arteries 1 month post-implantation. Representative cross-sections of the midgraft regions are shown. Implanted patches comprise the upper half of the vessel, magnification 20×.

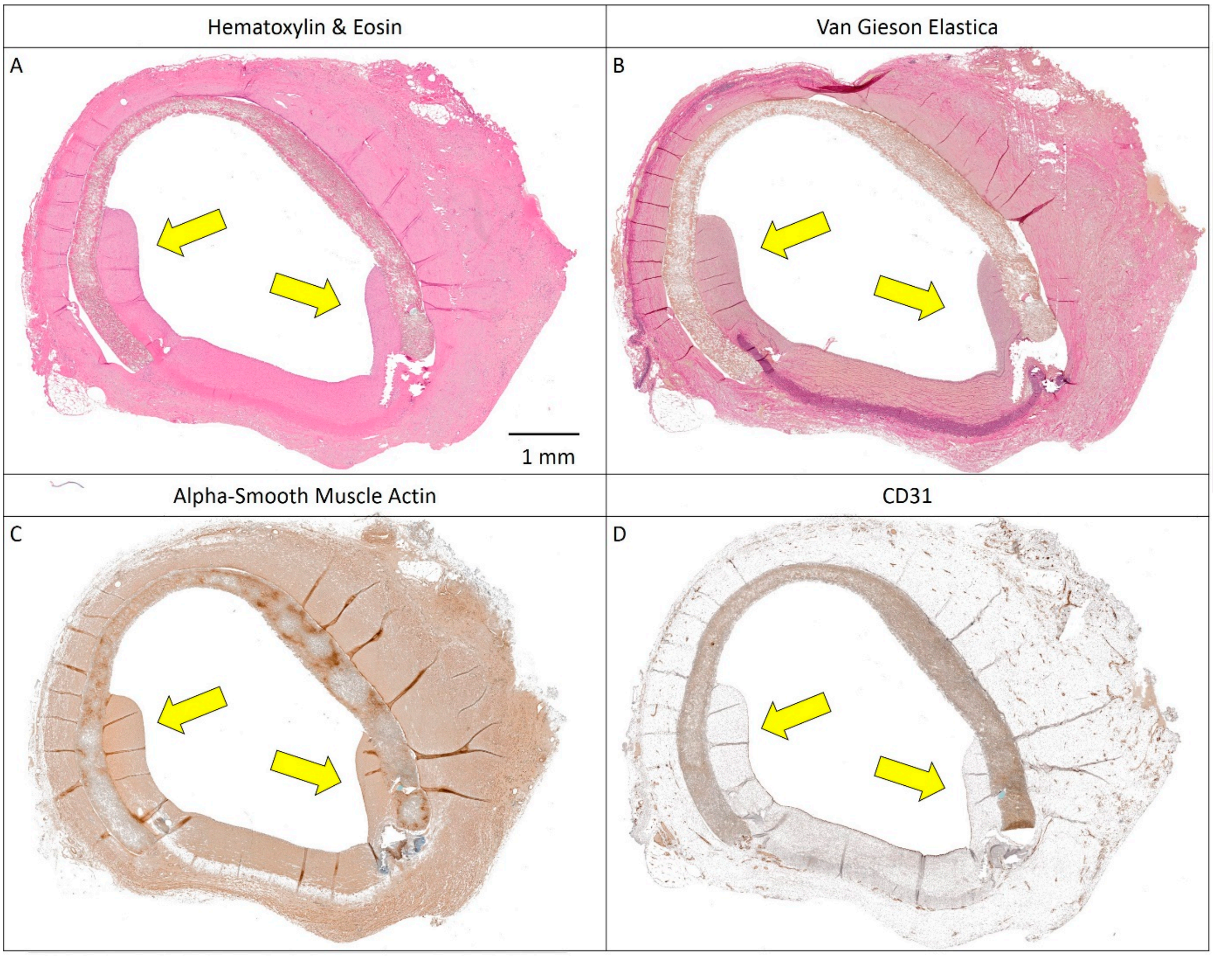

Supplementary Figure S13. Oversized vascular patch in a porcine carotid artery 1 month post-implantation - histology and immunohistochemistry. (**A**-**C**) Trans-anastomotic pannus overgrowth and (**D**) endothelial lining were observed from the native artery to the expanded polytetrafluoroethylene (ePTFE Gore® Propaten®) patch (yellow arrows). Representative cross-sections of the midgraft regions are shown. Implanted patches comprise the upper half of the vessel, magnification 20×.
